# An immunomodulatory decellularized pulp matrix hydrogel promotes vascularized dental pulp regeneration through angiogenic–odontogenic coupling

**DOI:** 10.1093/rb/rbag107

**Published:** 2026-06-02

**Authors:** Ping Yi, Runling Zeng, Zihao Gao, Yifan Zhao, Pei Li, Jiali Tan, Dongsheng Yu, Wei Zhao

**Affiliations:** Hospital of Stomatology, Sun Yat-sen University, Guangdong Provincial Key Laboratory of Stomatology, Guanghua School of Stomatology, Guangzhou, Guangdong, 510055, China; Hospital of Stomatology, Sun Yat-sen University, Guangdong Provincial Key Laboratory of Stomatology, Guanghua School of Stomatology, Guangzhou, Guangdong, 510055, China; Hospital of Stomatology, Sun Yat-sen University, Guangdong Provincial Key Laboratory of Stomatology, Guanghua School of Stomatology, Guangzhou, Guangdong, 510055, China; Department of Dentistry, Guangdong Women and Children Hospital, Guangzhou, Guangdong, 511442, China; Hospital of Stomatology, Sun Yat-sen University, Guangdong Provincial Key Laboratory of Stomatology, Guanghua School of Stomatology, Guangzhou, Guangdong, 510055, China; Hospital of Stomatology, Sun Yat-sen University, Guangdong Provincial Key Laboratory of Stomatology, Guanghua School of Stomatology, Guangzhou, Guangdong, 510055, China; Hospital of Stomatology, Sun Yat-sen University, Guangdong Provincial Key Laboratory of Stomatology, Guanghua School of Stomatology, Guangzhou, Guangdong, 510055, China; Hospital of Stomatology, Sun Yat-sen University, Guangdong Provincial Key Laboratory of Stomatology, Guanghua School of Stomatology, Guangzhou, Guangdong, 510055, China

**Keywords:** decellularized extracellular matrix, angiogenic–odontogenic coupling, macrophage polarization, vascularized dental pulp regeneration, pulp-capping materials, dental pulp tissue engineering

## Abstract

Maintaining the vitality of inflamed or exposed pulp is critical for long-term tooth preservation in clinical dentistry. Vital pulp therapy (VPT), achieved by placing bioactive materials over the exposed pulp tissue, aims to preserve pulp function and stimulate dentin–pulp healing. However, effective regeneration requires biomaterials capable of orchestrating early inflammatory resolution, immune modulation and coordinated angiogenic–odontogenic responses. Here, we engineered an injectable porcine-derived decellularized dental pulp matrix (pDPM) hydrogel with intrinsic immunomodulatory properties to promote vascularized dental pulp regeneration. The pDPM retained abundant pulp-specific extracellular matrix components and bioactive factors while displaying excellent biocompatibility. *In vitro*, solubilized pDPM stimulated PI3K/AKT pathway activation and promoted macrophage polarization toward the M2 phenotype. Using preconditioned Transwell co-culture models, pDPM was shown to facilitate angiogenic–odontogenic paracrine interactions, characterized by enhanced angiogenic activation of endothelial cells and promoted odontogenic differentiation of dental pulp stem cells. In a rat direct pulp-capping model, pDPM hydrogel treatment resulted in robust dentin bridge formation accompanied by enhanced neovascularization and a pro-regenerative immune microenvironment. Taken together, our findings demonstrate that pDPM integrates immunomodulatory and angiogenic–odontogenic to support vascularized dental pulp regeneration, highlighting its potential as a next-generation biomaterial for clinical VPT.

## Graphical Abstract

**Graphical Abstract:**
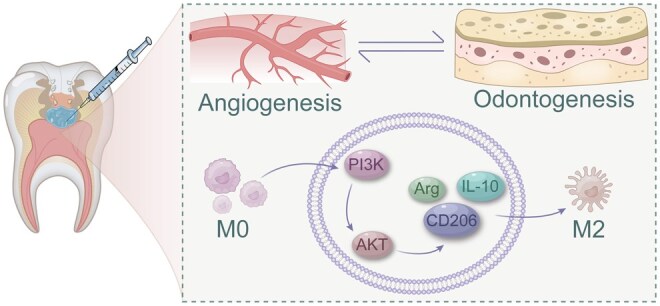

## Introduction

Pulpitis arising from caries, trauma or developmental disorders poses a major threat to long-term tooth preservation and prognosis [1]. Conventional root canal therapy has remained widely adopted in clinical practice for pulpitis, which could result in a complete pulp removal and sacrifices the immune function and possibility of re-infection of the pulp, leading to unsatisfactory long-term efficacy [2–4]. Hence, vital pulp therapy (VPT) has emerged as a promising alternative strategy for damaged pulp, aiming to preserve the residual vitality and function of dental pulp through minimally invasive intervention [5, 6]. The clinical efficacy of VPT critically depends on the performance of pulp-capping materials [7]. Nonetheless, conventional pulp-capping materials exhibit limited sealing performance and insufficient anti-inflammatory capacity, which may compromise pulp vitality and restrict dentin–pulp healing [8]. In addition, the confined root canal space and limited vascular supply pose significant challenges to effective tissue repair [9]. These challenges underscore the urgent need for next-generation pulp-capping materials capable of coordinating angiogenic and odontogenic responses while enabling minimally invasive delivery and conformal adaptation within the confined pulp space.

Decellularized extracellular matrix (dECM) is increasingly recognized as a bioactive, naturally derived scaffold that provides structural support while preserving intrinsic biochemical cues with minimal immunogenicity [10]. By retaining tissue-specific physicochemical signals and regulatory molecules, dECM facilitates cell–matrix interactions and orchestrates complex regenerative processes [11]. Within the dental field, decellularized dental pulp matrix (DPM) is of particular interest as it preserves dental pulp-specific proteins and growth factors essential for dentin–pulp regeneration [12–14]. DPM preserves dentin-specific extracellular matrix (ECM) proteins that support the proliferation and odontoblastic differentiation potential of dental pulp stem cells (DPSCs) [14]. A previous study further demonstrated that recellularization of DPM with DPSCs enhances angiogenesis-related marker expression *in vitro* and significantly improves vascularization *in vivo*, highlighting its intrinsic pro-angiogenic capacity [15]. Beyond serving as a biomimetic scaffold, DPM actively modulates the regenerative microenvironment by regulating macrophage polarization toward a reparative M2 phenotype [16], which is known to secrete pro-angiogenic and pro-odontogenic factors that facilitate vascularization and dentin formation. Collectively, these tissue-specific biochemical and immunomodulatory features position DPM as a promising scaffold for dentin repair, with the potential to support coordinated angiogenic–odontogenic regeneration.

While dentin regeneration is a primary goal of VPT, successful pulp repair critically relies on the coordinated formation of a functional vascular system that sustains tissue viability and homeostasis [17]. Increasing evidence indicates that odontogenesis and angiogenesis are tightly coupled processes, orchestrated through reciprocal paracrine signaling between odontoblast-lineage cells and endothelial cells [18, 19]. Endothelial-derived cues, particularly vascular endothelial growth factor (VEGF), enhance odontogenic gene expression in DPSCs. In turn, odontoblast-lineage cells secrete angiogenic mediators that recruit and activate endothelial cells, thereby reinforcing vascular–dentin coupling. Among the angiogenic regulators, VEGF and its primary receptor VEGFR2 play a central role in driving endothelial proliferation, migration and tube formation [20, 21]. A recent study identified a distinct vascular subtype in dental pulp, distinct from H-type vessels, in which VEGFR2 plays a central role in mediating angiogenesis–odontogenesis coupling, highlighting the molecular crosstalk underlying coordinated pulp regeneration [18]. However, whether DPM can actively orchestrate this coupling effect, rather than merely promoting angiogenesis or odontogenesis independently, remains unclear.

**Figure 1:**
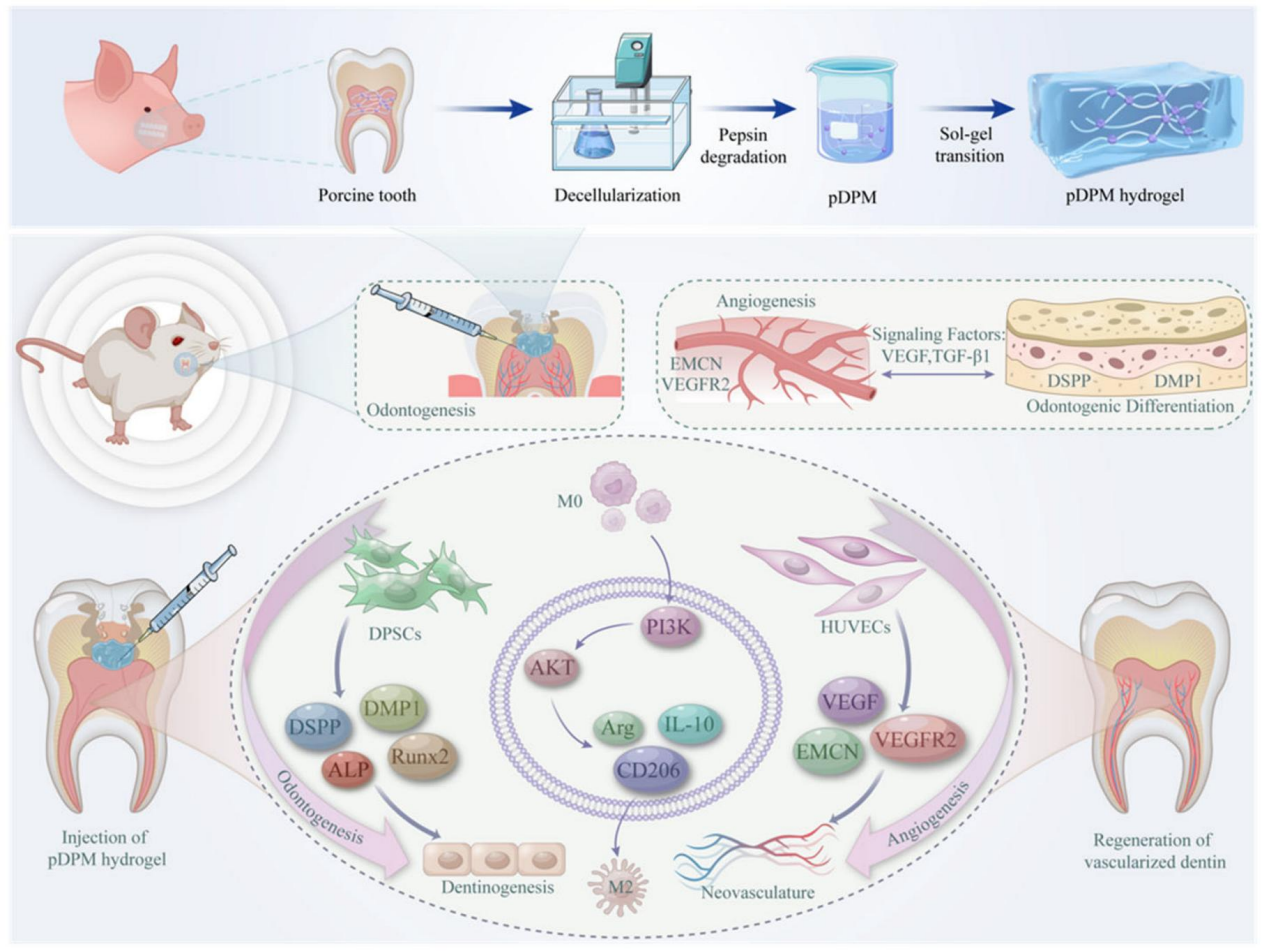
Schematic illustration of this study. pDPM hydrogel applied to exposed dental pulp promotes vascularized dental pulp regeneration. pDPM hydrogel derived from decellularized dental pulp regulates macrophage polarization via PI3K/AKT signaling, establishing a pro-regenerative immune microenvironment that enhances angiogenic–odontogenic coupling and promotes vascularized dental pulp regeneration.

In this study, we prepared a porcine-derived decellularized pulp matrix (pDPM) hydrogel and evaluated its ability to promote angiogenic–odontogenic coupling *in vitro* and *in vivo*. Specifically, we investigated the immunomodulatory effects of pDPM on macrophages and the paracrine interactions between human umbilical vein endothelial cells (HUVECs) and DPSCs, focusing on coordinated endothelial activation and odontoblastic differentiation. Based on these *in vitro* findings, we hypothesized that an acellular scaffold could enhance material-mediated vascularized dentin regeneration. This strategy aims to leverage immune regulation during biomaterial implantation to facilitate coordinated angiogenesis and dentin formation. The overall study design and proposed mechanism are illustrated in Figure 1.

## Materials and methods

All procedures involving animals were reviewed and authorized by the Animal Care and Use Committee of Sun Yat-sen University (approval no. SYSU-IACUC-2026-001539 and SYSU-IACUC-2026-001920). All *in vivo* studies were performed in compliance with the National Institutes of Health Guideline for the Care and Use of Laboratory Animals.

### Fabrication of DPM and pDPM hydrogel

Porcine mandibles aged about 6 months were obtained from the slaughterhouse in Guangzhou, China. Fresh dental pulp tissues were isolated from permanent teeth, sectioned into tiny strips, and washed with sterile phosphate-buffered saline (PBS). The tissues were first incubated in PBS containing 0.05% ethylenediaminetetraacetic acid (EDTA) and 0.02% trypsin. Samples were treated with 0.1% sodium dodecyl sulfate (Sigma-Aldrich, USA) for 48 h, followed by DNase (Takara, Japan) digestion for 1 h. Residual detergents were thoroughly eliminated by washing the samples in sterile PBS for no less than 48 h. The obtained DPM tissues were freeze-dried and milled into powder. The DPM powder was enzymatically digested in 0.01 M HCl containing pepsin (20 mg/mL) to generate the DPM pre-gel solution (DPM/pepsin = 10/1, *w/w*), for 48 h at room temperature and stored at −20°C.

To prepare pDPM-gel, the pre-gel solution was slowly titrated to approximately pH 7.4 using 1 M NaOH. Next, 10× PBS equivalent to one-ninth of the final volume was added carefully to achieve physiological acidity and salinity. Gelation was induced at 37°C for 30 min before the subsequent experiment.

### Evaluation of the decellularization process

Paraffin-embedded natural and DPM tissue were sectioned into slices for histological and immunohistology evaluation. Hematoxylin-eosin (H&E) staining was carried out to examine cellular and nuclear removal after decellularization. DNA quantification was measured with a PicoGreen dsDNA assay kit (Invitrogen, USA). Glycosaminoglycan (GAG) retention was quantified using Safranin O (Solarbio, Beijing, China) and a GAGs quantification kit (GENMED, USA). Masson’s trichrome staining was applied to visualize collagen distribution and structural organization. Collagen preservation was evaluated by collagen I immunostaining and hydroxyproline quantification using a commercial kit (Nanjing Jiancheng, China).

The microstructures of native dental pulp tissue, DPM, pDPM-gel and Collagen I hydrogel (Col I-gel) were investigated using a scanning electron microscope (SEM). Samples were fixed overnight, dehydrated with graded ethanol solutions, lyophilized, and platinum-coated prior to SEM characterization. Micromorphological characterization of native dental pulp tissue and DPM was viewed using an SEM (HITACHI, Japan) operated at an accelerating voltage of approximately 10 kV.

Rheological properties of pDPM-gel were characterized with a rheometer (TA Instruments, USA). Frequency sweep tests were performed at 37°C under 1% strain. Temperature and time sweep analyses were performed to assess the sol–gel transition and gelation kinetics, with temperature was increased from 4 to 40°C at 3°C/min, and a time sweep was conducted at 37°C under 1% strain and 1 Hz. Viscosity measurements were carried out over a shear rate ranging from 0.1 to 500 s^−1^ to assess shear-thinning behavior.

### Cell isolation, culture and co-culture models for angiogenic–odontogenic coupling

Human premolars or third molars without caries, periapical lesions, and restorations were obtained from 16- to 25-year-old healthy donors at the Hospital of Stomatology, Sun Yat-sen University, China. Dental pulps were aseptically extracted and minced using ophthalmic scissors. The tissue fragments were cultured in Dulbecco’s modified Eagle’s medium (DMEM, Hyclone, China) containing 10% fetal bovine serum (FBS), 100 U/mL penicillin and 100 μg/mL streptomycin under standard culture conditions (37°C, 5% CO_2_). The culture medium was refreshed every 2 days, and DPSCs at passages 3–5 were used. The HUVECs (ATCC, USA) were cultured in DMEM supplemented with 10% FBS and 1% penicillin–streptomycin. THP-1 (ATCC, USA) was cultured in RPMI 1640 medium supplemented with 10% FBS and 1% penicillin–streptomycin. For macrophage differentiation, THP-1 monocytes were exposed to 10 ng/mL phorbol-12-myristate-13-acetate (PMA) for 24 h, followed by incubation in PMA-free medium for another 24 h to allow phenotypic stabilization.

To dissect the directional crosstalk underlying angiogenic–odontogenic coupling, preconditioned Transwell co-culture models were established. For endothelial preconditioning, HUVECs were stimulated with pDPM for 24 h, enzymatically detached, and reseeded into the upper chambers of the Transwell insert (0.4 μm, Corning, USA). DPSCs were seeded in the lower chamber and were maintained in odontoblastic differentiation medium (α-MEM, 10% FBS, 50 μg/mL ascorbic acid, 10 mM β-glycerophosphate, and 100 nM dexamethasone) for subsequent evaluation of odontogenic differentiation. For odontogenic preconditioning, DPSCs were stimulated with pDPM for 24 h and subsequently reseeded into the upper Transwell inserts, while HUVECs were cultured in the lower chamber with complete culture medium. All Transwell co-culture experiments were conducted at 37°C with 5% CO_2_, and media were renewed every 2–3 days unless otherwise specified.

### Macrophage polarization, inflammatory modulation, and angiogenic–odontogenic factor analysis

Our previous research demonstrated that DPM effectively induced M2 polarization in RAW264.7 macrophages [16]. In this study, the influence of pDPM on THP-1-derived macrophage polarization was further investigated. THP-1-derived macrophages were generated as described in “Cell isolation, culture and co-culture models for angiogenic–odontogenic coupling.” Briefly, THP-1 monocytes were induced with PMA (10 ng/mL) for 24 h, followed by 24 h culture in PMA-free medium. The differentiated macrophages were subsequently exposed to pDPM for 24 h to evaluate its effects on macrophage polarization. Classical M1 and M2 polarization markers (CD86 and Arg-1/CD206) were used to characterize the polarization status. Immunofluorescence staining was performed for CD206, VEGF and TGF-β1 using primary antibodies diluted at 1:200 (Affinity, China). Quantitative polymerase chain reaction (qPCR) analysis was performed to evaluate the expression of *Arg-1*, *CD86*, *VEGF* and *TGF-β1*, representing M1 macrophages, M2 macrophages, angiogenesis and odontogenesis, respectively (Table S1). Protein levels of Arg-1, CD86, TGF-β1 and VEGF were further assessed by western blotting using primary antibodies diluted at 1:1000 (Affinity, China).

For inflammatory modulation analysis, THP-1-derived macrophages were first stimulated with LPS (100 ng/mL) for 12 h, followed by pDPM treatment for 24 h. Immunofluorescence staining was used to assess iNOS and CD206 using primary antibodies diluted at 1:200 (Affinity, China). Quantitative real-time PCR (qRT-PCR) was conducted to determine the expression level of *iNOS* and *IL-10*. Protein levels of iNOS and IL-10 were assessed by western blotting using primary antibodies diluted at 1:1000 (Affinity, China).

### Protein analysis of DPM and its regulatory mechanisms on macrophage polarization

Proteomic profiling of DPM (*n* = 3) was conducted by LC-MS/MS to characterize its peptide composition [22]. To assess donor-dependent variability in ECM constituents, three independent DPM batches were analyzed. Lyophilized samples were enzymatically digested with trypsin, and the resulting peptides were separated via a nano-LC system coupled to a timsTOF Pro mass spectrometer (Bruker Daltonics). Raw spectra were analyzed using MaxQuant against the Sus scrofa SwissProt database with a reverse decoy database, with the false discovery rate controlled below 1%. Functional annotation, including GO and KEGG pathway enrichment, was performed using DAVID, and ECM-related components were further categorized according to the MatrisomeDB repository [23].

To investigate the role of PI3K/AKT signaling in pDPM-mediated macrophage polarization, THP-1-derived macrophages were treated with pDPM in the presence or absence of LY294002 (10 μM). PI3K/AKT pathway activation was evaluated by western blotting of p-AKT and AKT after 24-h treatment. The involvement of PI3K/AKT signaling in macrophage polarization was further assessed by qRT-PCR analysis of CD86 and Arg-1. Protein expression was further evaluated by western blotting, with DMSO serving as the vehicle control.

### Cell proliferation

To evaluate the effects of endothelial-derived paracrine cues on DPSC proliferation, DPSCs were cultured alone or co-cultured with either untreated or pDPM-preconditioned HUVECs. A material-only control was included by adding pDPM to the upper insert without cells. DPSC proliferation was evaluated using an EdU staining. DPSCs were seeded at 1 × 10^4^ cells per well and maintained under the indicated conditions for 72 h. After staining with Adize488 and DAPI, fluorescence images from three randomly selected fields per group were captured using a confocal laser microscope (Zeiss LSM 980, Germany). FBS/DMEM medium (10%) was used as the blank control. The percentage of EdU-positive nuclei was quantified using ImageJ software.

### Cell migration and wound healing

The migration of cells was assayed using Transwell inserts with 8-μm pores (Corning, USA). DPSCs suspended in serum-free medium were seeded into upper chambers. According to the experimental grouping described above, DPSCs seeded in the upper inserts were allowed to migrate toward the lower chambers containing HUVECs with or without pDPM preconditioning. After 24 h of incubation, migrated cells were fixed and stained with crystal violet. FBS/DMEM medium (10% ) was used as the blank control.

For wound-healing assays, DPSCs were seeded into six-well plates. When cell confluence reached approximately 90%, a linear wound was generated using a sterile pipette tip. Wound closure was monitored at 0 and 24 h.

### Odontoblastic differentiation

To assess the paracrine influence of HUVECs and pDPM-conditioned media on odontogenic mineralization, DPSCs were cultured under the co-culture conditions described above. After 7 days, the alkaline phosphatase (ALP) activity of cells was detected by the ALP staining solution (Beyotime, China) and ALP activity detection kit. Following 14 days of culture, mineralized nodules were visualized using Alizarin Red staining, and the images were captured. For semi-quantitative analysis, the stain was quantified by measuring absorbance at 575 nm after destaining.

### Tube formation

To evaluate endothelial angiogenic responses under odontogenic cell–mediated paracrine regulation, HUVECs were cultured alone or exposed to paracrine cues derived from DPSCs with or without prior pDPM preconditioning, as described above. Tube formation was measured using Matrigel (Corning, USA). Briefly, after polymerization of Matrigel in 48-well plates, HUVECs were seeded onto the coated surfaces under the indicated conditions. Representative images were obtained using an inverted microscope, and tube numbers were quantified with ImageJ software.

### Quantitative real-time polymerase chain reaction

Gene expression levels associated with odontoblastic differentiation (*DMP1, DSPP, ALP* and *RUNX2*) at 14 days and angiogenic–odontogenic differentiation (*VEGF, VEGFR2, HIF-1α* and *EMCN*) at 3 days were analyzed using qRT-PCR. Cells were cultured under the Transwell co-culture conditions as described in “Cell isolation, culture and co-culture models for angiogenic–odontogenic coupling.” DPSCs were cultured in odontoblastic differentiation medium under Transwell co-culture conditions for 14 days prior to RNA extraction. For angiogenic assays, HUVECs were cultured under Transwell co-culture conditions for 3 days before analysis. Primer information is provided in Table S2.

### Western blot analysis

Protein expression levels of odontogenic markers (DMP1, DSPP, ALP and RUNX2) at 14 days and angiogenic-related genes (VEGF and VEGFR2) at 3 days were detected by Western blot analysis. Protein expression of DSPP, DMP1, RUNX2, ALP, VEGF and VEGFR2 was evaluated by western blotting using primary antibodies diluted at 1:1000.

### Immunofluorescence analysis of angiogenic markers in HUVECs under DPSC-mediated paracrine regulation

To investigate endothelial angiogenic responses under DPSC-mediated paracrine regulation, immunofluorescence staining was conducted to detect VEGF, VEGFR2 and EMCN using primary antibodies diluted at 1:200 (Affinity, China). After 3 days of treatment, fluorescence images were obtained using a confocal laser scanning microscope.

### *In vivo* biosafety evaluation by subcutaneous injection in mice

To evaluate the *in vivo* biosafety of pDPM hydrogel (pDPM-gel), healthy male mice (6–8 weeks old) were used. The pDPM-gel precursor solution was administered subcutaneously to the mice. At 7 days after injection, major organs were harvested for histological evaluation.

### Evaluation of reparative dentin formation in rat direct pulp-capping model with pDPM-gel

To evaluate the dentin regenerative capacity of pDPM-gel, a rat direct pulp-capping model was established. Eighteen male SD rats (8 weeks old, 300–350 g) comprising a total of 36 maxillary first molars were allocated into three groups: (1) blank control (PBS), (2) 5% Col I-gel, and (3) 5% pDPM-gel. They were operated according to the previously studied method [16]. Col I-gel, a widely used natural ECM-based biomaterial, was employed as a reference control for comparison with pDPM-gel [24, 25]. Following anesthesia with Zoletil 50, pulp exposure defects were created in the maxillary first molars using a 0.5 mm round bur under water cooling. The exposed pulp tissue was gently disrupted and irrigated before application of the designated formulation. The cavities were restored using glass ionomer cement (Fuji IX, Shizuoka, Japan). After 6 weeks, animals were euthanized, and maxillary specimens were harvested. Samples were decalcified in a 10% EDTA solution for 6 weeks and sectioned longitudinally into 5-μm slices for histological and immunohistochemical analyses.

### Statistical analysis

All statistical analyses were conducted using SPSS 25.0 software (SPSS Inc., Chicago, USA). Differences between groups were analyzed using Student’s *t*-test or one-way analysis of variance, where appropriate. A value of *P *< 0.05 was considered indicative of statistical significance.

## Results

### Characterization of DPM and pDPM-gel

**Figure 2:**
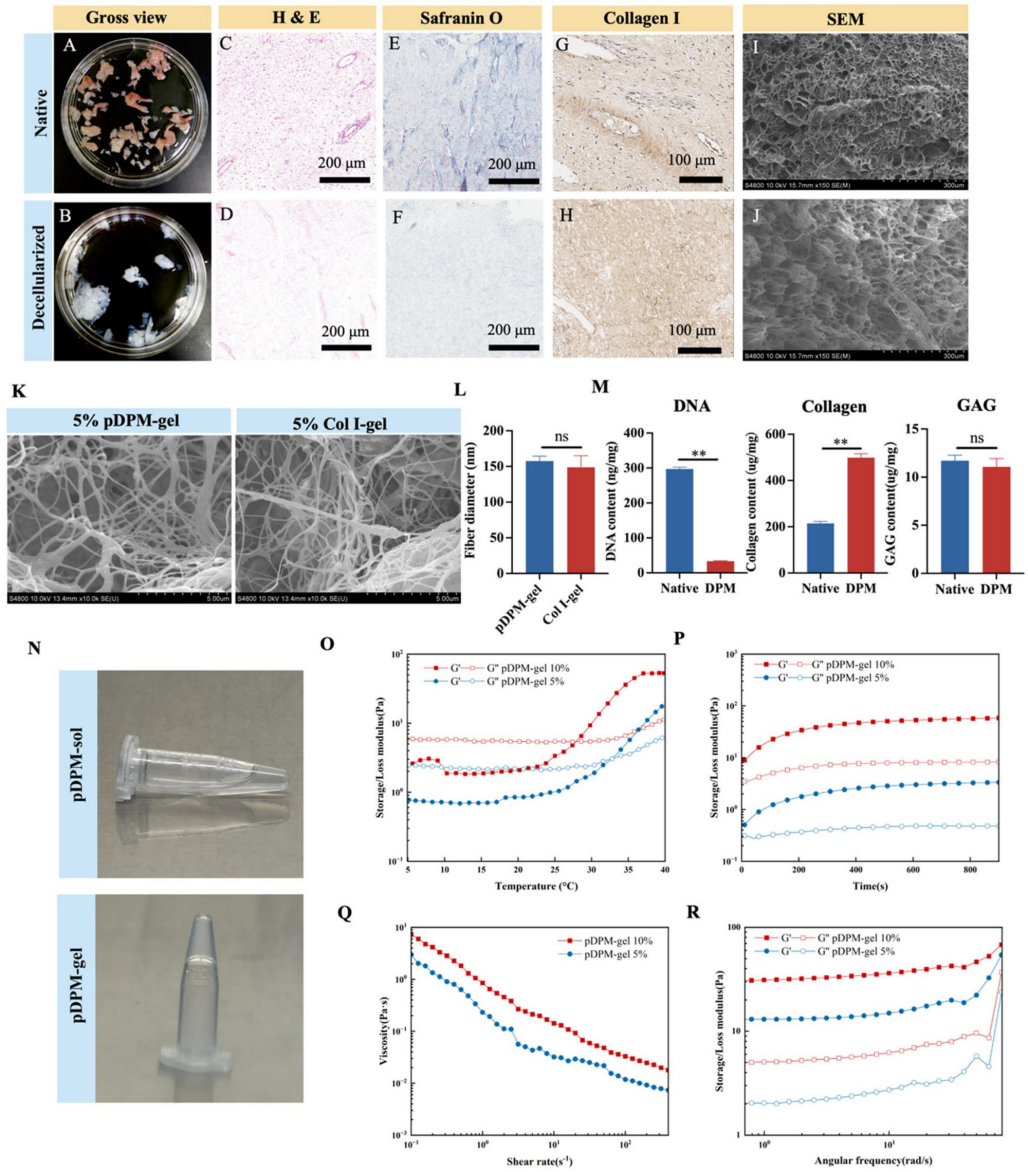
Fabrication and characterization of DPM and pDPM-gel. (**A**) Gross morphology of the fresh natural dental pulp tissue exhibiting a compact morphology. (**B**) Gross morphology of DPM showing a translucent and loose appearance. (**C, D**) H&E staining of native and decellularized dental pulp showing complete removal of cellular components while preserving the extracellular matrix structure. Scale bars = 200 μm. (**E, F**) Safranin O staining demonstrating the retention of glycosaminoglycans (GAGs). Scale bars = 200 μm. (**G, H**) Immunohistochemical staining for collagen I confirms preservation of collagen in the DPM. Scale bars = 100 μm. (**I, J**) SEM revealed that the decellularized matrix maintained a porous microstructure and ECM fiber orientation like native dental pulp. Scale bars = 300 μm. (**K, L**) SEM images and corresponding fiber diameter of the nanofibrous structure of the pDPM-gel and col I-gel. Scale bars = 5 μm. (**M**) Quantitative evaluation of the DNA elimination, collagen preservation and GAG retention. (**N**) Schematic illustration of the sol-to-gel transition process of pDPM under physiological conditions. (**O**) Temperature sweep showing thermoresponsive gelation of pDPM hydrogel (G’: storage modulus; G”: loss modulus). (**P**) Time sweep showing rapid gelation kinetics (G’ > G”). (**Q**) Viscosity measurement showing shear-thinning behavior. (**R**) Frequency sweep showing stable viscoelastic properties (G’ > G”). Data are presented as mean ± SD (*n* = 3). ns, not significant; *P < 0.05, **P < 0.01.

Following the decellularization process, the originally pink and intact dental pulp tissues (Figure 2A) became white and translucent in appearance and loose in consistency (Figure 2B). H&E staining demonstrated effective removal of cellular components while preserving the overall ECM architecture in DPM (Figure 2C and D). Safranin O staining and collagen I confirmed the retention of GAGs and collagen within the DPM (Figure 2E–H). SEM observation showed that DPM preserved a microarchitecture comparable to native dental pulp (Figure 2I and J). Moreover, the pDPM-gel exhibited a nanofibrous architecture with fiber morphology and diameter distribution like those of Col I-gel (Figure 2K and L). Quantitative analyses demonstrated a marked reduction in DNA content following decellularization, while collagen content and GAG retention were largely preserved in the DPM (Figure 2M). In addition, a schematic illustration was provided to depict the sol-to-gel transition process of pDPM, showing its injectable fluid state at low temperature and gel formation at physiological temperature (Figure 2N).

To further evaluate the physicochemical properties of pDPM-gel, rheological analyses were performed. Temperature sweep analysis demonstrated that pDPM-gel exhibited thermoresponsive gelation behavior, with the storage modulus (G’) increasing markedly as the temperature rose from 4 to 37°C and surpassing the loss modulus (G”), indicating a transition from a sol to a gel state (Figure 2O). Time sweep analysis at 37°C revealed rapid gelation kinetics, with G’ increasing sharply and reaching a plateau within approximately 300–400 s (∼5 min), suggesting the establishment of a stable viscoelastic network (Figure 2P). Viscosity measurements further showed that pDPM-gel exhibited a typical shear-thinning property, supporting its injectability for minimally invasive delivery (Figure 2Q). In addition, frequency sweep analysis confirmed the viscoelastic stability of the hydrogel, with G’ consistently higher than G” across the tested frequency range, indicating a stable gel structure (Figure 2R).

### pDPM induces M2 polarization and enhances angiogenic–odontogenic factor expression in THP-1-derived macrophages

**Figure 3:**
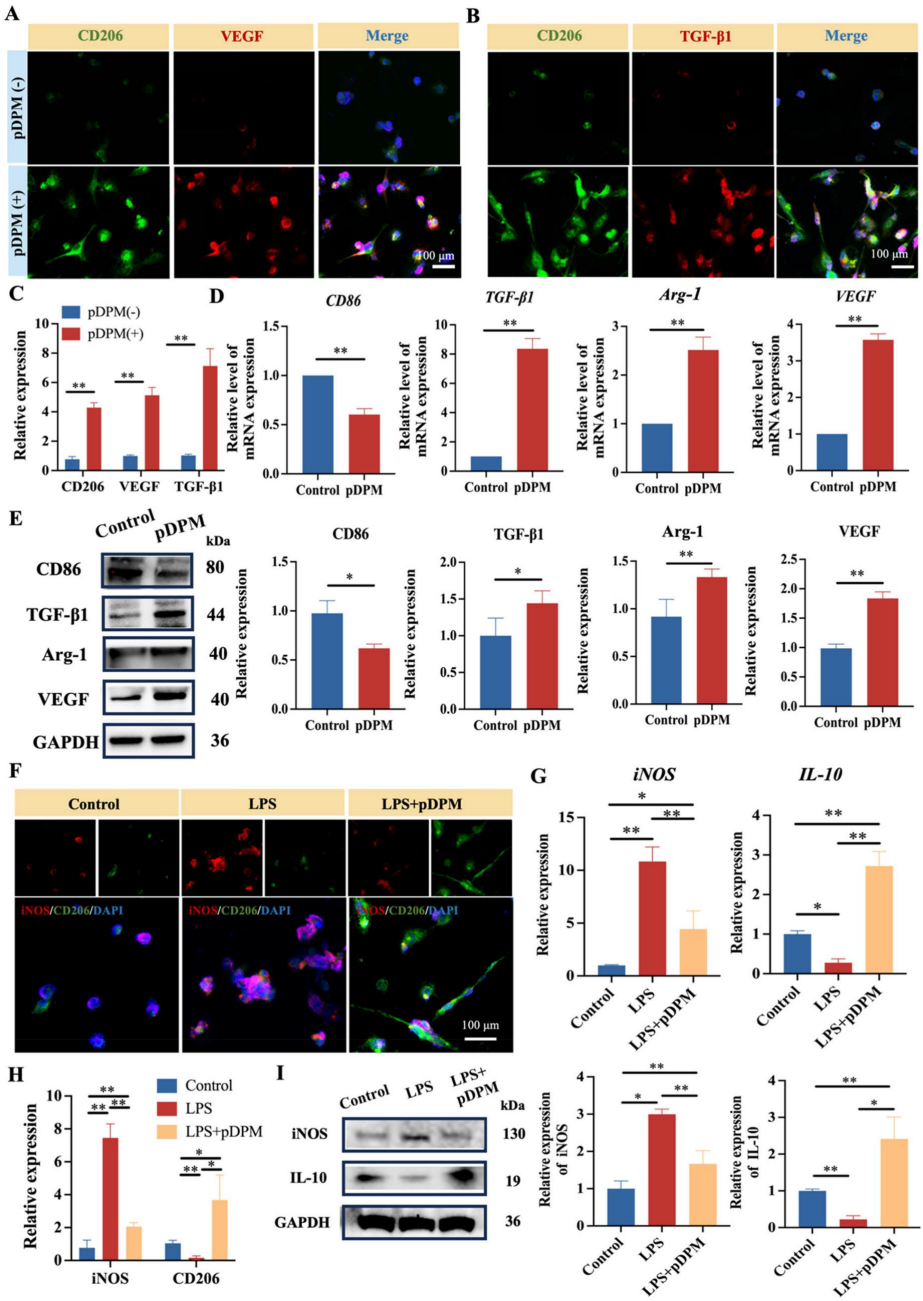
(**A**) Representative immunofluorescence images showing the percentage of CD206-positive and VEGF-positive cells in the control and pDPM groups. (**B**) Representative immunofluorescence images showing TGF-β1 expression in THP-1-derived macrophages following pDPM treatment compared with the control group. (**C**) Semi-quantitative immunofluorescence analysis of CD206, VEGF and TGF-β1 expression. (**D**) qRT-PCR analysis of *CD86, arg-1, TGF-β1*and *VEGF* mRNA expression normalized to GAPDH. (**E**) Western blotting and densitometric analysis of CD86, arg-1, TGF-β1 and VEGF protein levels. (**F**) Representative immunofluorescence staining of CD206 (green) and iNOS (red) expression in Control, LPS and LPS + pDPM groups. (**G**) qRT-PCR evaluate of *iNOS* and *IL-10* mRNA expression normalized to GAPDH. (**H**) Semi-quantitative analysis of iNOS and CD206 fluorescence intensity. (**I**) Western blotting and densitometric analysis of iNOS and IL-10. Scale bars: 100 μm. Data are presented as mean ± SD (*n* = 3). ns, not significant; *P < 0.05, **P < 0.01.

Immunofluorescence staining showed that pDPM treatment significantly increased the expression of the M2-associated marker CD206 in THP-1-derived macrophages, indicating robust M2 polarization. In addition to phenotypic polarization, pDPM-treated macrophages exhibited significantly elevated expression of VEGF and TGF-β1, indicating the acquisition of a pro-regenerative secretory profile encompassing both angiogenic and odontogenic-related factors (Figure 3A–C). Consistent with the immunofluorescence findings, qRT-PCR analysis corroborated these observations, revealing a significant downregulation of CD86, alongside upregulation of Arg-1. Notably, the transcription levels of VEGF and TGF-β1 were also significantly increased in response to pDPM stimulation (Figure 3D). Western blot further confirmed these trends, demonstrating a decrease in CD86 expression accompanied by a concomitant increase in Arg-1, TGF-β1 and VEGF levels (Figure 3E).

To further evaluate whether pDPM retains its immunomodulatory activity under an inflammatory microenvironment, an LPS-stimulated macrophage model was generated. Immunofluorescence staining demonstrated that pDPM markedly reduced iNOS expression while increasing CD206 expression compared with the LPS group (Figure 3F and H). qRT-PCR analysis showed decreased *iNOS* levels, together with increased *IL-10* expression following pDPM treatment (Figure 3G). Western blot analysis further confirmed reduced iNOS protein expression and elevated IL-10 levels in the pDPM-treated group (Figure 3I).

Collectively, these results demonstrate that pDPM not only promotes M2 polarization of THP-1-derived macrophages but also enhances their expression of angiogenic and odontogenic-related factors, supporting a pro-regenerative macrophage phenotype potentially conducive to vascularized dental pulp regeneration.

### Proteomic profiling reveals an immunoregulatory ECM microenvironment in DPM associated with PI3K/AKT-dependent macrophage polarization

**Figure 4:**
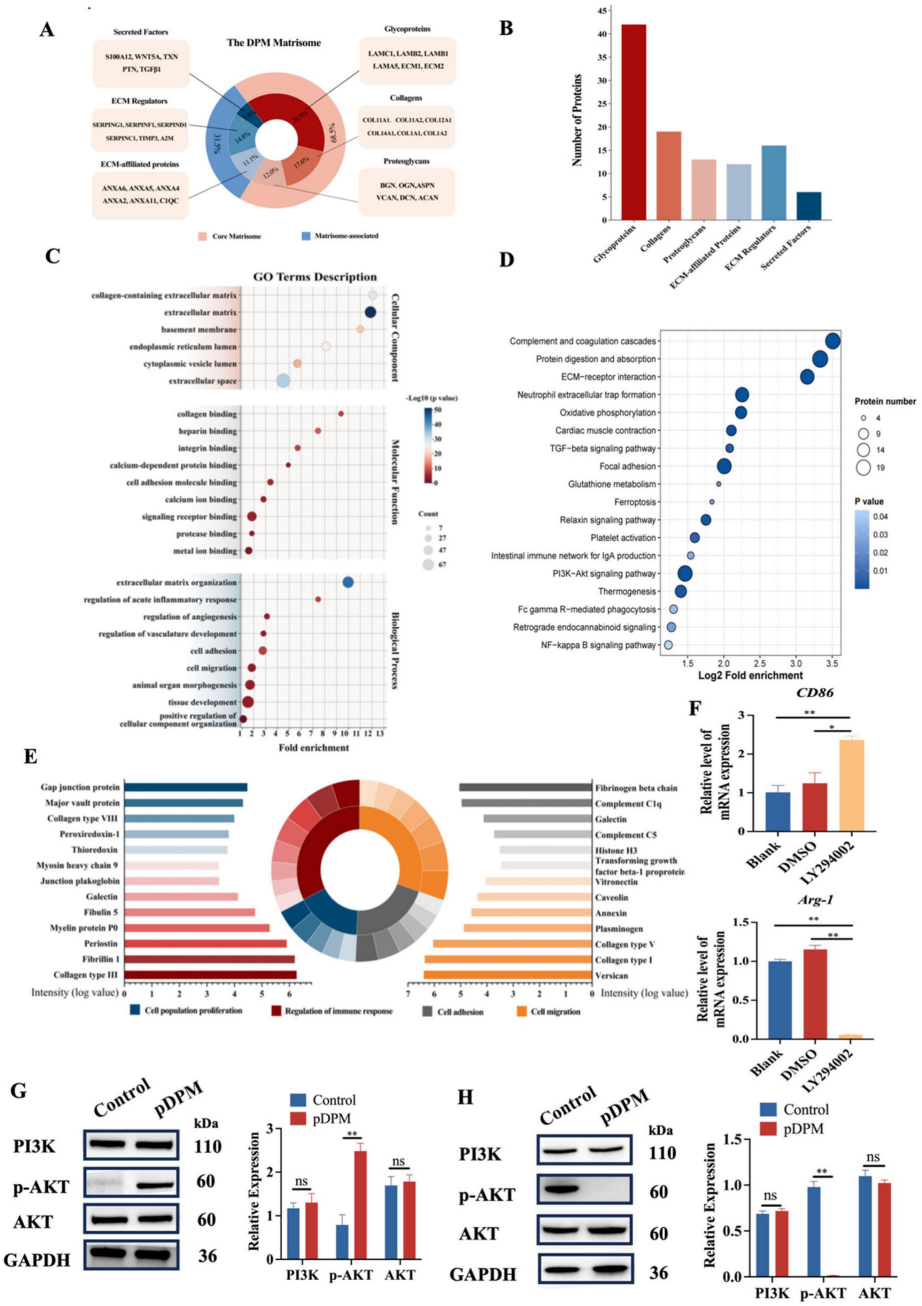
Proteomic analysis of the DPM and PI3K/AKT-dependent regulation of macrophage M2 polarization. (**A**) Schematic workflow of LC-MS/MS-based proteomic analysis of DPM. (**B**) Quantitative distribution of proteins across different matrisome categories. (**C**) GO enrichment results of identified DPM proteins. (**D**) KEGG pathway analysis of the DPM proteins. (**E**) Functional distribution of DPM proteins related to cell adhesion, migration, proliferation and immune modulation. (**F**) qRT-PCR analysis of macrophage polarization markers after pharmacological inhibition of PI3K/AKT signaling with LY294002. (**G**) Western blot analysis showing increased p-AKT in macrophages following pDPM treatment. (**H**) Western blot confirming LY294002 suppressed p-AKT induced by pDPM, whereas the DMSO had no effect. Data are presented as mean ± SD (*n* = 3). ns, not significant; *P < 0.05, **P < 0.01.

To elucidate the intrinsic bioactive composition of DPM, a comprehensive LC-MS/MS proteomic analysis identified 260 distinct matrisome proteins, reflecting the preservation of the native dental pulp ECM signature (Figure  4A and B). These proteins were classified into the core matrisome—comprising collagens (COL1A1, COL1A2, COL11A2), glycoproteins (LAMA5, LAMB2, LAMC1), and proteoglycans (BGN, OGN, DCN) and matrisome-associated components, including ECM regulators (SERPINB9, TIMP3), ECM-affiliated proteins (ANXA2, C1QC), and secreted factors (TGF-β1, WNT5A, PTN). Core matrisome proteins are crucial for ECM integrity and cell–matrix adhesion, while associated proteins actively modulate immune signaling and tissue repair. Of note, TGF-β1 and WNT5A, highly represented in the secretome, are well-known mediators of macrophage polarization [26, 27], suggesting that DPM provides not only a structural but also an immunoregulatory matrix microenvironment.

GO analysis indicated that DPM proteins were mainly associated with ECM modeling, regulation of angiogenesis and immune-related biological processes (Figure 4C). At the molecular function level, enrichment was observed for collagen binding, integrin binding, and cell adhesion molecule binding, whereas cellular component analysis indicated strong association with collagen-containing ECM and basement membrane structures (Figure 4C). Among the ECM-associated proteins, fibronectin 1 (FN1), type I collagen, and type V collagen were prominently represented and have been previously reported to promote M2 polarization (Figure 4E) [28, 29]. These findings suggest that DPM retains a bioactive ECM repertoire that supports immunomodulation and establishes a pro-regenerative microenvironment conducive to vascularized dentin regeneration.

KEGG pathway enrichment further demonstrated significant involvement of these proteins in ECM-associated and immune-regulatory pathways (Figure 4D). These pathways are closely involved in cell–matrix communication, immune regulation, and tissue regeneration. Notably, the PI3K/AKT signaling pathway showed prominent enrichment, suggesting its potential involvement in DPM-mediated immunomodulation.

To further elucidate the molecular mechanisms underlying pDPM-mediated immunomodulation, activation of the PI3K/AKT signaling pathway was examined by western blotting. Compared with the control group, pDPM treatment markedly upregulated the level of phosphorylated AKT (p-AKT) in macrophages, while total AKT expression remained unchanged (Figure 4G). To verify the functional involvement of this pathway, the pan-PI3K inhibitor LY294002 was applied to block AKT phosphorylation. qRT-PCR analysis revealed that inhibition of PI3K/AKT signaling significantly downregulated M2-associated gene marker Arg-1 while increasing M1-associated gene markers CD86 (Figure 4F). Consistently, western blotting confirmed that LY294002 effectively suppressed pDPM-induced p-AKT levels, whereas the DMSO control group had no detectable impact on either macrophage polarization markers or AKT activation (Figure  4H). Together, these results indicate that pDPM establishes a pulp-specific immunoregulatory ECM microenvironment that promotes macrophage M2 polarization through PI3K/AKT signaling.

### pDPM-stimulated endothelial paracrine signaling enhances DPSCs proliferation, migration and odontogenic differentiation

**Figure 5:**
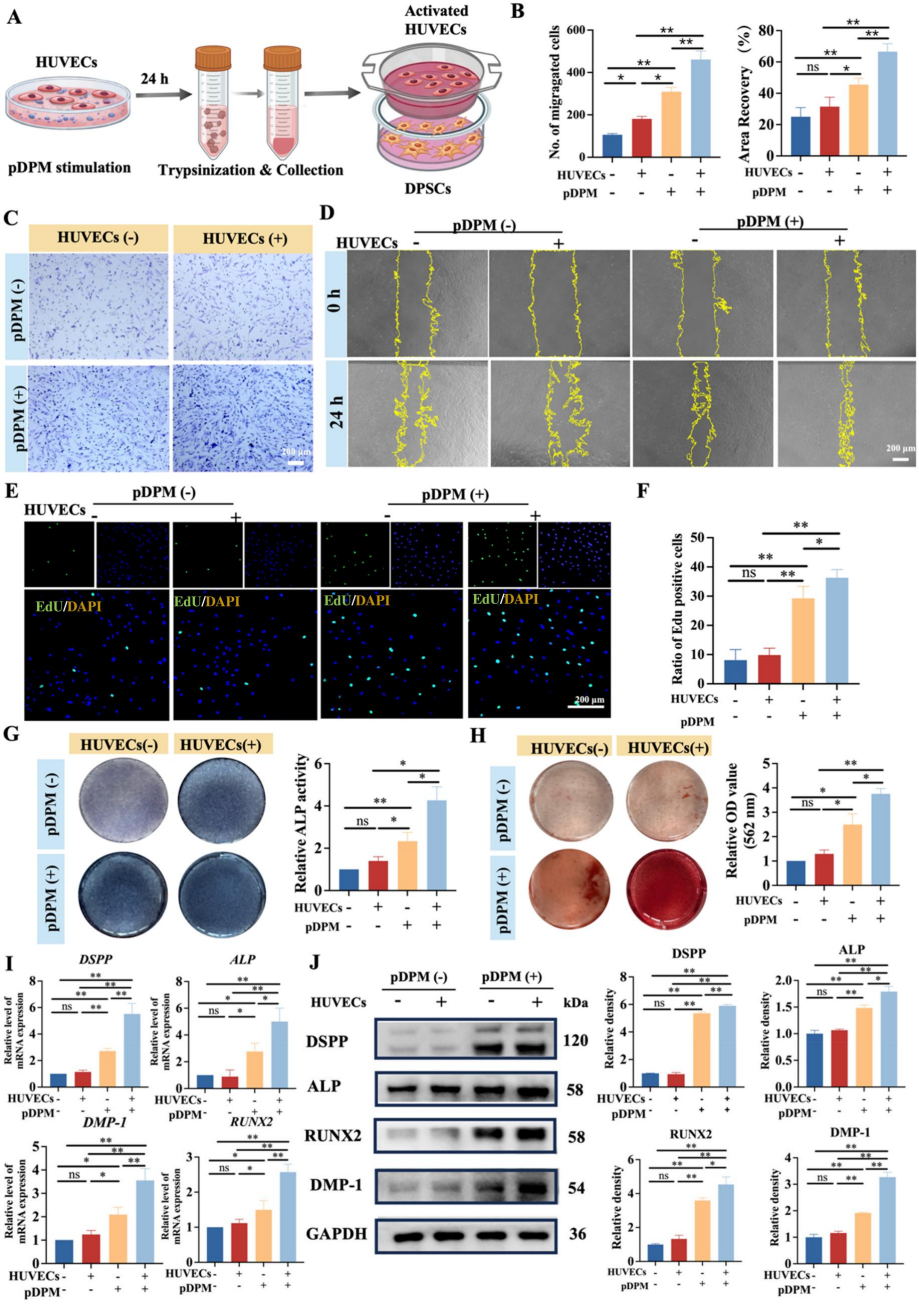
pDPM-Stimulated endothelial paracrine signaling promotes DPSCs activation and odontogenic differentiation. (**A**) Schematic illustration of a pDPM-preconditioned Transwell co-culture model for evaluating endothelial paracrine regulation of DPSCs. (**B**) Quantitative analysis of migrated DPSCs numbers per field in Transwell assays and scratch closure rate in migration assays. (**C**) Representative images of DPSCs migration in the Transwell assay. (**D**) Representative scratch wound-healing images. (**E**) Representative confocal images of EdU-stained DPSCs after 72 h in different conditioned media. (**F**) Quantification of EdU-positive cells. (**G**) Gross images and quantitative analysis of ALP staining of DPSCs. (**H**) Gross images of alizarin red staining and semi-quantitative analysis of calcium deposition. (**I**) qRT-PCR analysis of odontogenic gene expression levels. (**J**) Western blot analysis and quantification of odontogenic proteins in DPSCs. Scale bars: 200 μm. Data are presented as mean ± SD (*n* = 3). ns, not significant; *P < 0.05, **P < 0.01.

Given the role of endothelial paracrine signaling in mesenchymal cell regulation, we next investigated whether pDPM modulates endothelial-mediated functional response in DPSCs. A preconditioned Transwell co-culture model was established, as illustrated in Figure 5A. In the Transwell assay, a markedly greater number of DPSCs migrated through the membrane in the pDPM-HUVECs group, forming dense clusters on the lower surface, while only sparse cells were observed in the control (Figure 5B and C). Similarly, in wound-healing assay, scratch gaps in the pDPM-HUVECs group showed substantial closure at 24 h, with spindle-shaped cells actively moving into the denuded area (Figure 5B and D). EdU incorporation assays revealed a marked increase in DPSCs’ proliferative activity when co-cultured with pDPM-preconditioned HUVECs (Figure 5E and F). These findings indicate that pDPM-activated endothelial paracrine signaling effectively enhances both the proliferative and migratory capacities of DPSCs.

Beyond cellular activation, DPSCs differentiation was substantially enhanced by pDPM-stimulated HUVECs’ paracrine signaling. After 7 days, DPSCs co-cultured with the pDPM-preconditioned HUVECs group exhibited intense ALP staining, with deep purple coloration across most of the culture surface, whereas only scattered positive areas were observed in the blank control (Figure 5G). Alizarin Red S staining at 14 days showed abundant and densely distributed mineralized nodules forming confluent clusters in the pDPM-HUVECs group, in contrast to the sparse mineral deposition detected in the control group (Figure 5H). At the molecular level, qRT-PCR analysis at day 14 revealed that the mRNA expression of odontogenic markers including *DSPP*, *DMP1*, *RUNX2*, and *ALP* was significantly upregulated in the pDPM-HUVECs group (Figure 5I). Protein upregulation of DSPP, DMP1, RUNX2 and ALP was further verified by western blotting (Figure 5J). These results indicate that pDPM-stimulated HUVEC paracrine signaling robustly promotes matrix mineralization and odontogenic commitment of DPSCs.

### pDPM enhances HUVEC migration and HUVEC angiogenesis through endothelial–DPSC interactions *in vitro*

**Figure 6:**
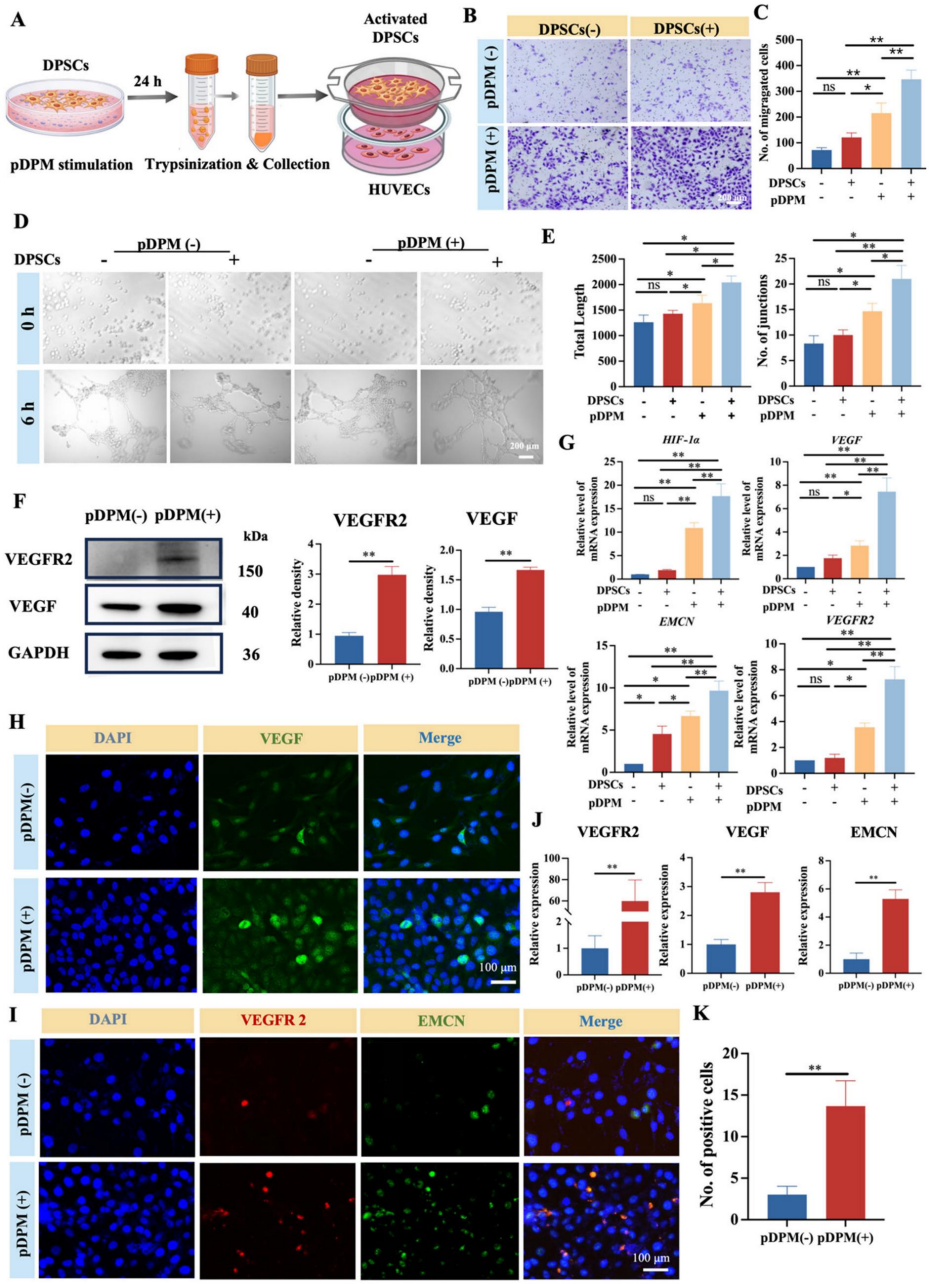
pDPM-stimulated endothelial paracrine crosstalk enhances endothelial activation and establishes angiogenic–odontogenic coupling *in vitro*. (**A**) Schematic illustration of the pDPM-preconditioned co-culture model used to evaluate DPSC-mediated regulation of endothelial angiogenic responses. (**B**) Representative images and of HUVECs migration (scale bar: 200 μm). (**C**) Quantification of migrated HUVECs per field. (**D**) Tube formation images (scale bar: 200 μm). (**E**) Quantification of total tube length and the number of closed loops. (**G**) qRT-PCR analysis of angiogenesis-related gene expression. (**F**) Western blot analysis and quantification of odontogenic proteins in HUVECs. (**H**) Immunofluorescence images showing VEGF expression (scale bar; 100 μm). (**I**) Immunofluorescence images showing VEGFR2 and EMCN expression (scale bar: 100 μm). (**J**) Semi-quantitative immunofluorescence analysis of VEGF, VEGFR2, and EMCN expression. (**K**) Quantification of VEGFR2+EMCN per field. Scale bars: 100 μm. Data are presented as mean ± SD (*n* = 3). ns, not significant; *P < 0.05, **P < 0.01.

To evaluate whether pDPM indirectly promotes endothelial angiogenic responses through interactions with DPSCs, a preconditioned co-culture model was established, as illustrated in Figure 6A. HUVECs exposed to the pDPM-DPSCs group exhibited a pronounced migratory response, with a greater number of cells traversing the membrane and displaying an elongated, spindle-like morphology compared with control groups (Figure  6B and C).

Endothelial angiogenic activation was further evaluated. When co-cultured with pDPM-preconditioned DPSCs, HUVECs rapidly organized into interconnected capillary-like networks within 6 h (Figure 6D and E). qRT-PCR analysis revealed elevated expression of key angiogenic and endothelial-associated genes (*VEGF, VEGFR2, HIF-1α* and *EMCN*) in the pDPM-HUVECs group (Figure 6G). Consistently, VEGF and VEGFR2 protein levels were elevated in co-cultured cells under pDPM stimulation (Figure 6F). Immunofluorescence staining further confirmed enhanced endothelial activation, as evidenced by intensified VEGF, VEGFR2, and EMCN signals and an increased number of VEGFR2^+^EMCN^+^ endothelial cells (Figure 6H–K).

These results collectively demonstrate that pDPM promotes endothelial recruitment, accelerates tube formation and activates angiogenic gene expression, thereby establishing a pro-angiogenic microenvironment favorable for vascularized dental pulp regeneration.

### pDPM-gel promotes reparative dentin formation *in vivo*

**Figure 7:**
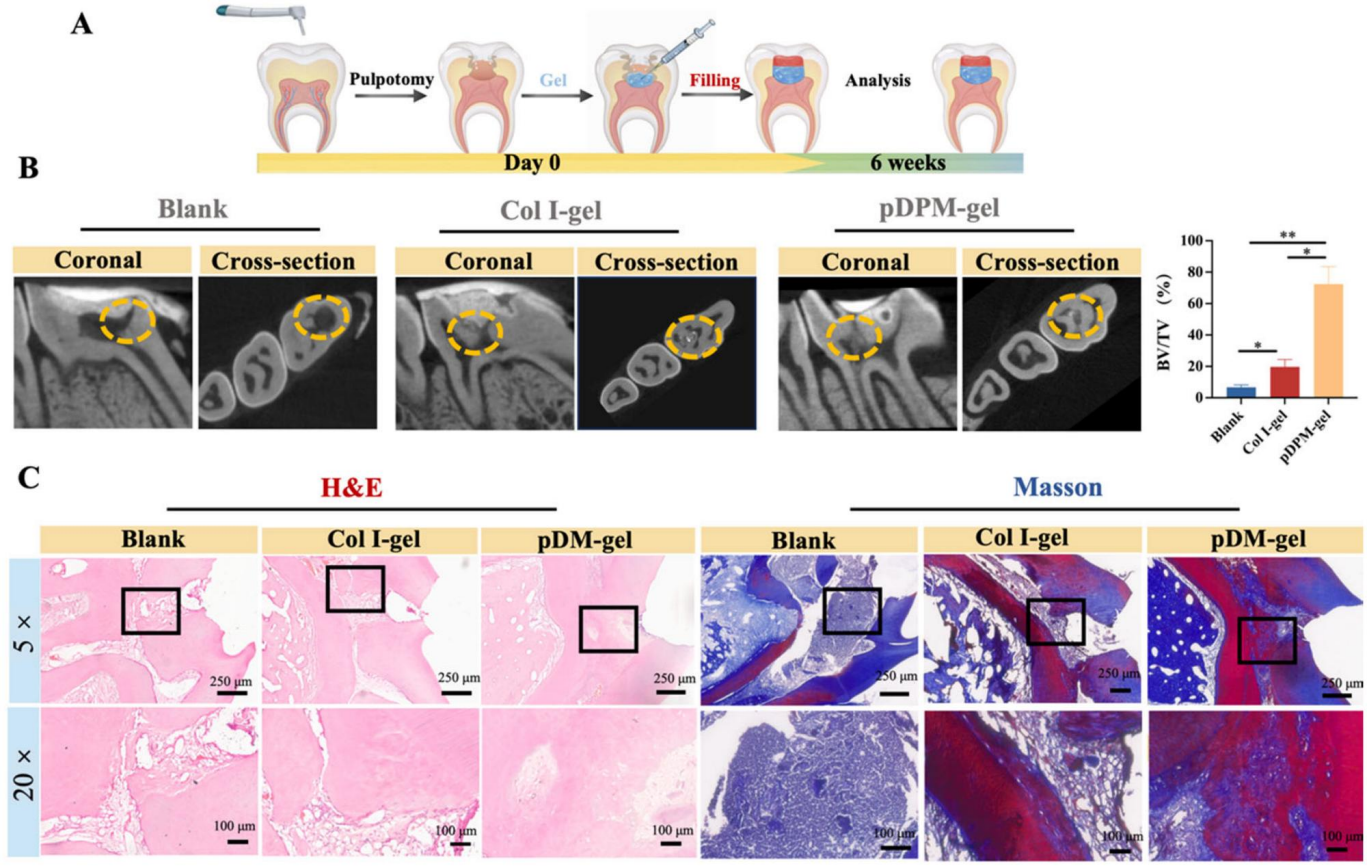
pDPM Hydrogel promotes reparative dentin formation in a rat direct pulp-capping model. (**A**) The flowchart of the animal experiment. (**B**) Representative micro-CT images and quantitative analysis of newly formed mineralized tissue in each group. The indicated regions indicate dentin bridge formation at the pulp-dentin interface. (**C**) Representative histological evaluation by H&E and Masson’s trichrome staining of pulp exposure sites in each group, showing reparative dentin formation.

To evaluate the regenerative performance of the pDPM-gel *in vivo*, a rat direct pulp-capping model was established, followed by *in situ* injection of PBS, 5% Col-gel or 5% pDPM-gel (Figure 7A). Samples were harvested 6 weeks postoperatively for radiographic and histological evaluation. Micro-CT reconstruction revealed minimal mineralized tissue formation in the PBS group, with the exposed pulp space remaining largely unbridged (Figure 7B). The Col-gel group exhibited limited and discontinuous hard tissue deposition at the pulp-dentin interface, indicating only modest reparative activity. In contrast, the pDPM-gel group showed a markedly thicker and more continuous dentin-like bridge, indicative of enhanced reparative dentin formation.

Histological analysis further corroborated these findings. H&E and Masson’s trichrome staining (Figure 7C) confirmed pDPM-gel promoted new dentin formation. The PBS group displayed persistent pulp exposure and inflammatory cell infiltration, whereas the Col-gel group showed partial coverage by loosely organized reparative tissue. The pDPM-gel group exhibited the formation of a compact, well-organized dentin structure with collagen deposition.

### pDPM-gel enhances vascular reconstruction and M2 macrophage polarization *in vivo*

**Figure 8:**
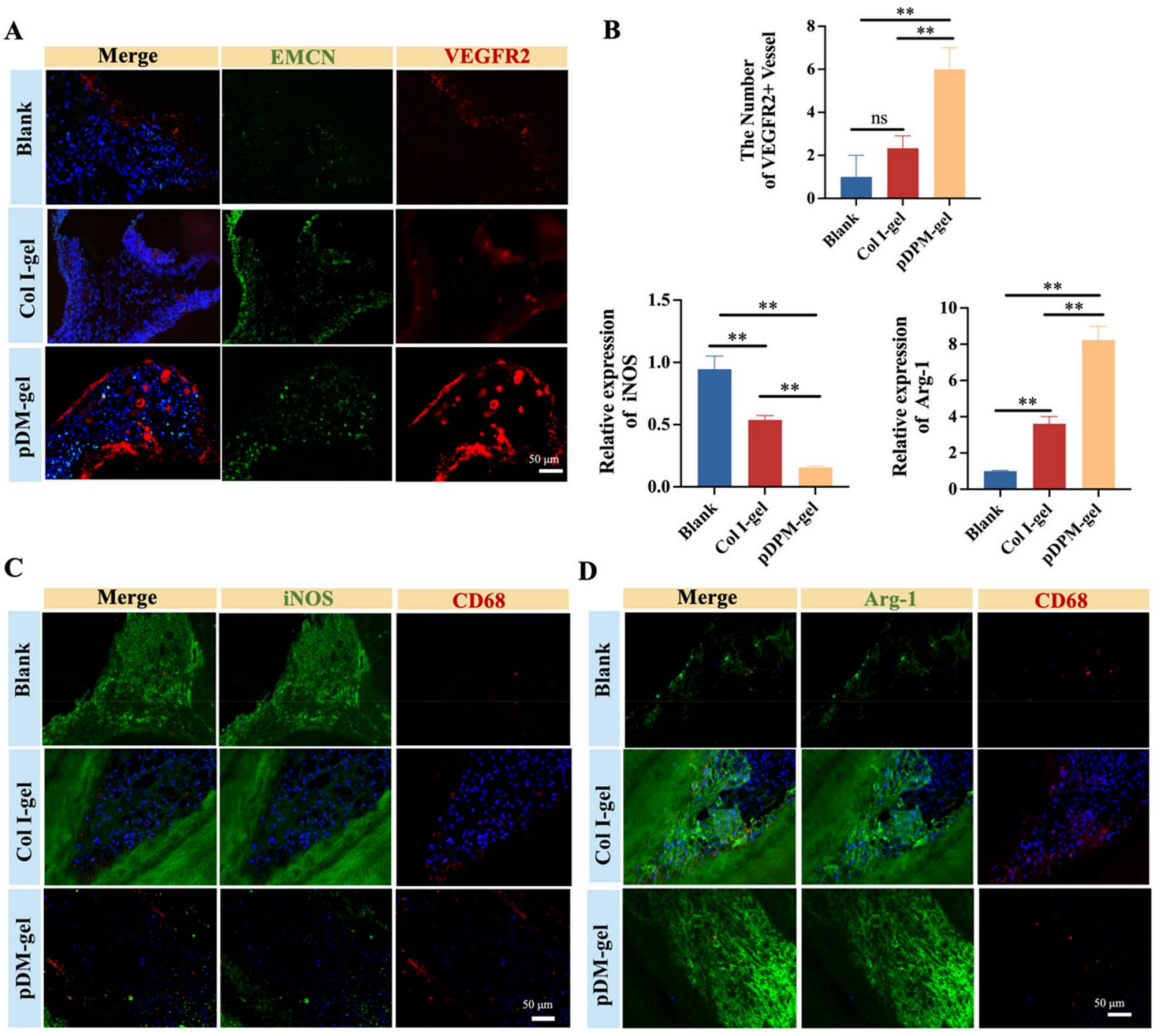
pDPM hydrogel enhances vascular reconstruction and M2 macrophage polarization *in vivo*. (**A**) Representative immunofluorescence images showing VEGFR2 and EMCN expression in reparative dentin *in situ*. (**B**) Semi-quantification of VEGFR2-positive area, iNOS and arg-1. (**C-D**) Representative immunofluorescence images of CD68/iNOS and CD68/arg-1 for macrophage polarization in reparative dentin *in situ*. Scale bars: 200 μm. Data are presented as mean ± SD (*n* = 3). ns, not significant; *P < 0.05, **P < 0.01.

To determine whether enhanced dentin regeneration was accompanied by vascular reconstruction, immunofluorescence staining for endothelial markers was performed. Compared with the PBS and Col-gel groups, the pDPM-gel-treated group exhibited markedly higher expression of VEGFR2 and EMCN, indicating robust endothelial recruitment and neovascularization (Figure 8A and B). To further determine whether the distinct regenerative outcomes were associated with macrophage polarization, immunofluorescence staining for CD68/iNOS (M1) and CD68/Arg-1 (M2) was performed. CD68 is a marker of *in situ* macrophages (Figure 8B–D). CD68-positive cells were detected in all samples, indicating the pivotal role of macrophages in dental repair. The intensity of iNOS-positive staining was significantly higher in the PBS group, whereas implantation of Col-gel moderately reduced iNOS-positive staining. In contrast, Arg-1-positive staining intensity was significantly higher in the pDPM-gel group, with a marked decrease observed in iNOS-positive cells. These results demonstrate that pDPM-gel promotes vascularized dental pulp regeneration *in vivo* by enhancing endothelial reconstruction and facilitating macrophage polarization toward a pro-regenerative M2 phenotype. In addition, no evident pathological abnormalities were observed in major organs at 7 days after subcutaneous injection of pDPM-gel in mice, indicating no obvious histopathological abnormalities in major organs at the examined time point (Figure S2).

## Discussion

dECM scaffolds have emerged as bioinstructive platforms that retain tissue-specific signaling while minimizing immunogenicity [30]. Rather than serving merely as structural templates, dECM actively regulates immune remodeling and cell fate decisions [31–33]. In the context of VPT, successful dentin–pulp regeneration requires coordinated resolution of inflammation and timely vascular reconstruction [34, 35]. Here, we identify porcine-derived DPM as an immunoregulatory ECM scaffold that integrates immune modulation with angiogenic–odontogenic coupling.

Consistent with previous studies demonstrating that tissue-specific dECM retains native microarchitectures and regenerative signaling cues [36], our decellularization strategy maintained key structural features and matrix components. This preservation may support the retention of adhesion-related motifs and biophysical cues characteristic of the native pulp ECM. The pDPM-gel further recapitulated this structure within a three-dimensional matrix environment while exhibiting thermoresponsive gelation and shear-thinning behavior, enabling injectability and stable retention within the pulp cavity. In the *in vivo* procedure, the hydrogel was allowed to undergo initial gelation for approximately 5 min before placement of the overlying glass ionomer, which was sufficient to support material retention at the exposure site. Rather than serving as a load-bearing or sealing material, the hydrogel functions as a viscoelastic intracavitary matrix that adapts closely to cavity walls and supports the sealing performance of the overlying restoration. These properties suggest that pDPM is structurally compatible with current clinical protocols in VPT while providing additional biological functionality [37, 38].

Proteomic profiling revealed enrichment of immunoregulatory components, suggesting its ability to shape the inflammatory microenvironment that influences downstream regenerative outcomes. The identified matrisome-associated secretome was enriched in TGF-β1, WNT5A, FN1, and multiple collagen subtypes, all of which have been extensively implicated in immunoregulation, wound healing, and macrophage M2 polarization [39–42]. Although targeted quantification of individual growth factors was not performed, the regenerative activity of pDPM may be mediated by the combined effects of retained matrix-associated proteins, glycosaminoglycans, structural ligands, and tissue-specific microenvironmental cues rather than isolated soluble factors alone [43, 44]. Such a composite bioactive profile provides a molecular basis for the immunomodulatory capacity of pDPM, suggesting that the bioactive cues retained within the decellularized matrix may actively instruct macrophage phenotypic responses. KEGG analysis highlighted dominant enrichment of the PI3K/AKT pathway. Subsequent functional experiments confirmed its essential role in pDPM-induced macrophage polarization. These findings are consistent with established roles of PI3K/AKT signaling in anti-inflammatory macrophage programming [45–47]. The abundance of ECM ligands such as fibronectin and collagen may contribute to this effect by engaging integrin receptors and promoting downstream AKT activation [41, 48, 49]. Collectively, these findings support an active immunomodulatory role for pDPM mediated by ECM–immune signaling interactions.

Building upon this immunoregulatory conditioning, successful pulp capping requires coordinated vascular reconstruction and odontogenic activation [50, 51]. In this study, pDPM polarized macrophages toward an M2 phenotype and modulated the microenvironment to a pro-regenerative state. This immune regulation was associated with enhanced angiogenic–odontogenic crosstalk, including increased angiogenic activation in HUVECs and promoted odontogenic differentiation of DPSCs. pDPM-induced macrophages also upregulated TGF-β1 and VEGF expression, suggesting that their paracrine cues contribute to this coordinated response [52]. This was accompanied by increased expression of VEGF, VEGFR2 and other angiogenesis-related markers in HUVECs, together with elevated odontogenic marker expression in DPSCs following pDPM preconditioning. VEGFR2-expressing endothelial subtypes have been identified as key mediators of this coupling in dental pulp [18], supporting a potential role for VEGF/VEGFR2-associated endothelial responses in the coordinated vascular and odontogenic effects of pDPM. These observations indicate that pDPM supports coordinated vascular and odontogenic responses during dentin–pulp regeneration.

*In vivo* studies further established the translational potential of pDPM-gel as a clinically adaptable biomaterial. Compared with the PBS and Col-gel groups, pDPM-gel significantly enhanced dentin bridge thickness, continuity, and mineralized tissue formation, aligning with features of high-quality reparative dentin. Immunofluorescence analysis showed marked increases in VEGFR2-positive vessels in the pDPM-treated pulp, indicating robust endothelial recruitment and active neovascularization during dentin regeneration. These findings are consistent with previous reports demonstrating that angiogenesis is indispensable for pulp wound healing and reparative dentin by ensuring oxygen, nutrient supply, and progenitor cell support within the confined pulp space [53]. Notably, M2-polarized macrophages in the pDPM-gel group exhibited a spatial distribution closely aligned with regions of active-matrix remodeling and dentin bridge formation, while PBS-treated pulps displayed diffuse inflammatory cell infiltration dominated by iNOS-positive macrophages. This pattern is consistent with prior studies identifying M2 polarization as a key determinant of inflammation resolution and regenerative progression in dental pulp tissues [54, 55]. Together, the coordinated enrichment of reparative macrophages, endothelial activation, and organized dentin matrix deposition indicates tightly coupled immune, vascular, and matrix remodeling in pDPM-treated pulps. Such coordinated immune modulation and vascular remodeling may be critical for durable dentin–pulp regeneration [56], supporting pDPM-gel as a promising scaffold for advanced VPT.

In summary, this study identifies a porcine-derived decellularized pulp matrix as an immunoregulatory ECM scaffold that promotes PI3K/AKT–mediated M2 polarization and angiogenic–odontogenic coupling to achieve vascularized dental pulp regeneration. Future work should further define the specific matricellular cues underlying its immunomodulatory effects and explore strategies to optimize the regenerative microenvironment for improved therapeutic outcomes.

## Conclusion

This study demonstrates pDPM functions as an immuno-instructive extracellular scaffold that orchestrates vascularized dental pulp regeneration. pDPM directs macrophage polarization toward a reparative M2 phenotype via PI3K/AKT signaling, leading to secretion of key cytokines, which in turn enhances angiogenic–odontogenic paracrine interactions. As a direct pulp-capping material, pDPM-gel significantly enhanced vascularized dental pulp formation. These findings highlight immune–vascular–odontogenic coupling as a key mechanism in dentin–pulp regeneration and support pDPM-gel as a promising biomaterial for advanced VPT.

## Supplementary Material

rbag107_Supplementary_Data
