# Supplementary material for: An immunomodulatory decellularized pulp matrix hydrogel promotes vascularized dental pulp regeneration through angiogenic–odontogenic coupling
Source: Regen Biomater. 2026 Jun 2;13:rbag107. doi: 10.1093/rb/rbag107 (PMC13287997; doi:10.1093/rb/rbag107)
Supplement: rbag107_Supplementary_Data [file rbag107_supplementary_data.zip › Supplemental material5.25(3).docx]

Supplemental Information

**An Immunomodulatory Decellularized Pulp Matrix Hydrogel Promotes Vascularized Dental Pulp Regeneration Through Angiogenic-Odontogenic Coupling**

Ping Yi ^1,^ ^†^, Runling Zeng ^1,^ ^†^, Zihao Gao^1,^ ^†^, Yifan Zhao^2^, Pei Li^1,^, Jiali Tan ^1,^ ^∗^, Dongsheng Yu ^1,^ ^∗^ and Wei Zhao^1, ∗^

^1^. Hospital of Stomatology, Sun Yat-sen University, Guangdong Provincial Key Laboratory of Stomatology, Guanghua School of Stomatology, Guangzhou, Guangdong, China.

^2^. Department of Dentistry, Guangdong Women and Children Hospital, Guangzhou, Guangdong, China.

^†^. These three authors contributed equally to this work.

*. Corresponding Authors.

E-mail: tanjiali@mail.sysu.edu.cn (J. Tan), yudsh@mail.sysu.edu.cn (D. Yu), zhaowei3@mail.sysu.edu.cn (W. Zhao)

**Table S1**. Primers for qPCR analysis of THP-1.

| Target gene | Primer sequences |
| --- | --- |
| *GAPDH* | Forward: CATCCGTAAAGACCTCTATGCCAAC |
|  | Reverse: ATGGAGCCACCGATCCACA |
| *CD86* | Forward: TGGTGACATGTTCCTGGAGG |
|  | Reverse: GGTGATGGTGAGAAGGAGGT |
| *Arg-1* | Forward: GGTGTCCTGTGCTGTGTTTG |
|  | Reverse: GAGATGGGATGTCTTCAGTGAA |
| *VEGF* | Forward: TGCAGGCTGCTGTAACGATG |
|  | Reverse: GGAACAAGGCTCACAGTGATTT |
| *TGF-β1* | Forward: CAGCAACAATTCCTGGCGATA |
|  | Reverse: GTTGAGCCGTGTTGAGCAGAA |
| *iNOS* | Forward: GAGCCAGGCCACCTCTATGT |
|  | Reverse: GTCCTCGACCTGCTCCTCAT |
| *IL-10* | Forward: GACTTTAAGGGTTACCTGGGTTG |
|  | Reverse: TCACATGCGCCTTGATGTCTG |

**Table S2**. Primers for qPCR analysis of DPSCs and HUVECs.

| Target gene | Primer sequences |
| --- | --- |
| *GAPDH* | Forward: TCTCCTCTGACTTCAACAGCGACA |
|  | Reverse: CCCTGTTGCTGTAGCCAAATTCGT |
| *DMP-1* | Forward: GAGCAGTGAGTCATCAGAAGGC |
|  | Reverse: GAGAAGCCACCAGCTAGCCTAT |
| *DSPP* | Forward: CAACCATAGAGAAAGCAAACGCG |
|  | Reverse: TTTCTGTTGCCACTGCTGGGAC |
| *ALP* | Forward: CCTCCTCGGAAGACACTCTG |
|  | Reverse: GCAGTGAAGGGCTTCTTGTC |
| *RUNX2* | Forward: CCACTGAACCAAAAAGAAATCCC  Reverse: GAAAACAACACATAGCCAAACGC |
| *VEGF* | Forward: CAAACCTCACCAAAGCCAGC |
|  | Reverse: CGCCTTGGCTTGTCACATTTTT |
| *VEGFR2* | Forward: AGCCAGAGTGTGTTGGTTGT |
|  | Reverse: AGTAGCCAGGAAATCGGACT |
| *HIF1-α* | Forward: GCCGCAGGACACACAAGT |
|  | Reverse: CAGGGCTGTGTCGACTGTT |
| *EMCN* | Forward: AATACCAGGCATCGTGTCAGT |
|  | Reverse: CCACTTCATGTTTTGGTGTTGTC |


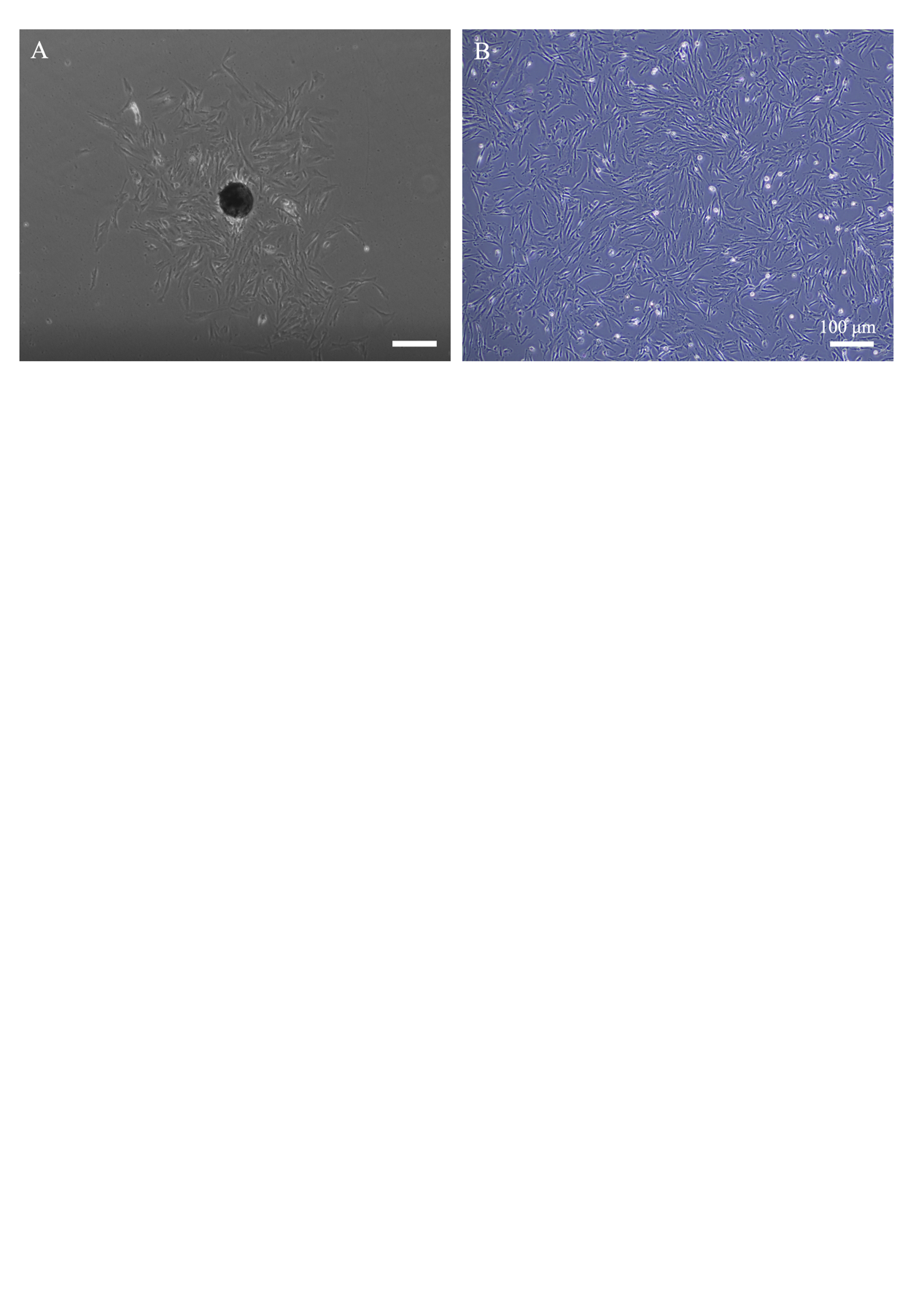


**Fig S1.** Characterization and proliferation of DPSCs. 3 days of primary DPSCs (A) and DPSCs at P3 (B).


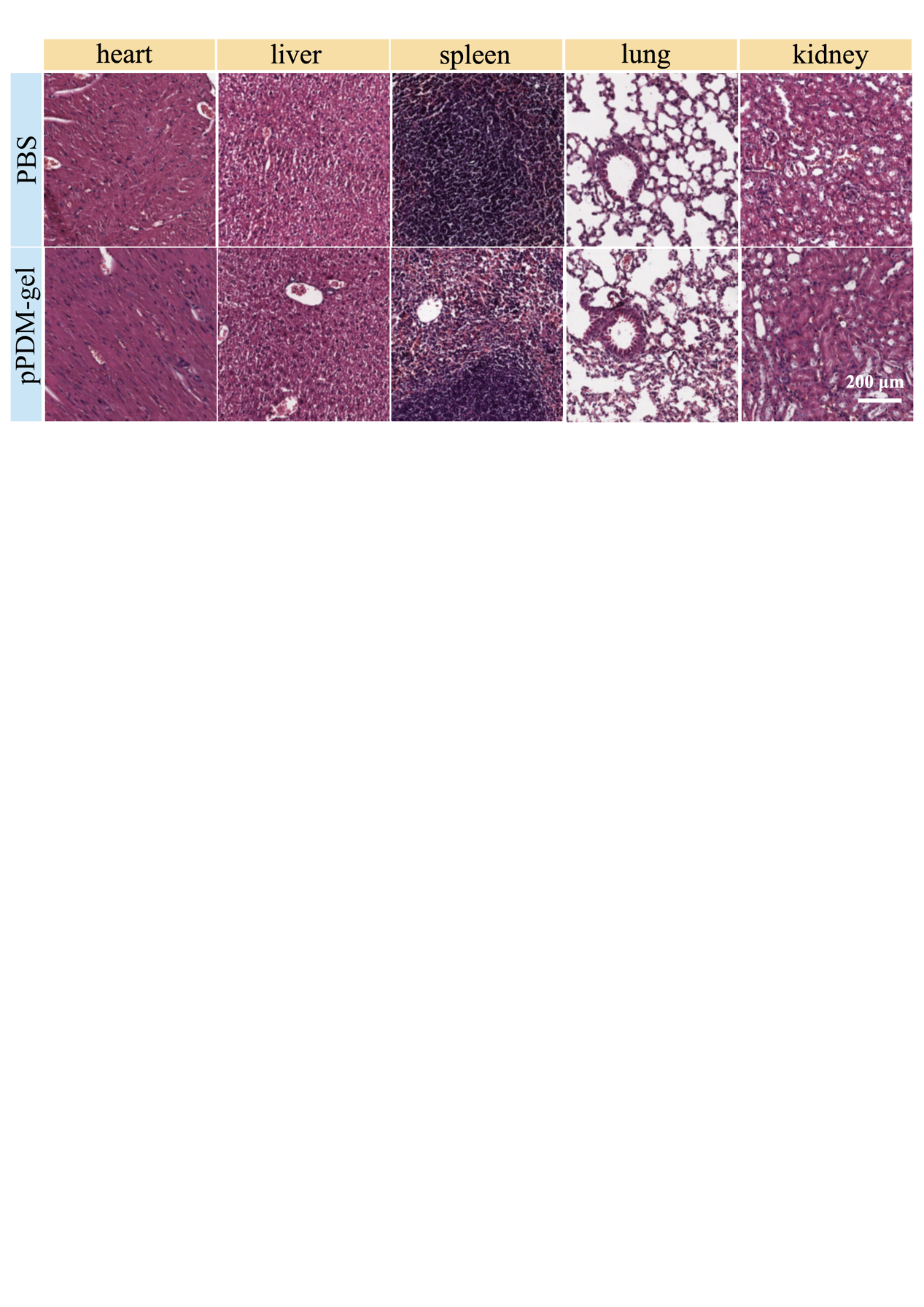


**Fig S2.** Histological evaluation of major organs following subcutaneous injection of pDPM-gel in mice.
