# Supplementary material for: An immunomodulatory decellularized pulp matrix hydrogel promotes vascularized dental pulp regeneration through angiogenic–odontogenic coupling
Source: Regen Biomater. 2026 Jun 2;13:rbag107. doi: 10.1093/rb/rbag107 (PMC13287997; doi:10.1093/rb/rbag107)
Supplement: rbag107_Supplementary_Data [file rbag107_supplementary_data.zip › Response Letter5.7(1).docx]

**Response Letter**

Dear Editor,

Thank you for the opportunity to revise and resubmit our manuscript (RB-2026-186). We sincerely appreciate the insightful and constructive comments provided by you and the reviewers, which have been invaluable in improving the quality and clarity of our work.
We have carefully considered all the comments and have thoroughly revised the manuscript accordingly. Each concern has been addressed in a point-by-point manner, with additional experiments and analyses performed. All revisions have been clearly indicated in red in the revised manuscript for ease of review.
We believe that these substantial revisions have significantly strengthened the rigor, clarity, and overall quality of the manuscript. We hope that the revised version meets the standards of the journal and will be suitable for publication.

In addition, we would also like to note a minor correction to the author information. Due to an oversight on our part during manuscript preparation, the name “Runlin Zeng” was incorrectly provided in the original submission. The correct name is “Runling Zeng.” This has been updated in the revised manuscript. No other author details, including email address and affiliations, are affected.
Thank you again for your time and consideration. We look forward to your feedback.

Best regards,
Sincerely yours,
Wei Zhao
Hospital of Stomatology, Sun Yat-sen University, Guangdong Provincial Key Laboratory of Stomatology, Guanghua School of Stomatology, Guangzhou, Guangdong, China.
Email: zhaowei3@mail.sysu.edu.cn

Dongsheng Yu
Hospital of Stomatology, Sun Yat-sen University, Guangdong Provincial Key Laboratory of Stomatology, Guanghua School of Stomatology, Guangzhou, Guangdong, China.
Email: yudsh@mail.sysu.edu.cn

Jiali Tan
Hospital of Stomatology, Sun Yat-sen University, Guangdong Provincial Key Laboratory of Stomatology, Guanghua School of Stomatology, Guangzhou, Guangdong, China.
Email: tanjiali@mail.sysu.edu.cn

**Response to Reviewer #1**

*Comment：*This study applied a dental pulp-derived decellularized extracellular matrix hydrogel as a pulp capping adjuvant material. Through the synergistic regulation of HUVECs and DPSCs, as well as the modulation of macrophages toward a pro-reparative phenotype, dentin repair and angiogenesis were achieved. The study demonstrates certain innovation.

*Response：*Thank you for your precious time to review our manuscript and provide valuable comments. These comments are helpful for improving our article. All the authors have extensively discussed these comments and have tried their best to modify the manuscript. Based on your suggestions, we have refined the terminology, supplemented methodological details, improved data presentation, and incorporated additional analyses to strengthen the mechanistic and translational relevance of the manuscript.

*Comment 1*: Is the term "Vascularized dentin regeneration" appropriate? In the context of vascularized bone regeneration, "Vascularized bone regeneration" refers to bone tissue rich in blood vessels. However, in dental pulp tissue, regenerated dentin does not contain vascular structures; dentin and blood vessels represent two distinct structural domains. The intended meaning of the authors appears to be the synergistic functional coordination between odontogenesis and angiogenesis. It is recommended to modify the expression accordingly.

*Response 1*: We appreciate this important comment. We fully agree that the term “vascularized dentin regeneration” may be misleading, as dentin itself is an avascular tissue and does not contain vascular structures. As correctly pointed out, our intention was to describe the coordinated interaction between angiogenesis and odontogenesis rather than implying vascularization within dentin itself. Accordingly, we have carefully revised the terminology throughout the manuscript to improve conceptual accuracy and clarity. The misleading expression has been removed, and “**angiogenic–odontogenic coupling**” is now consistently used to describe the coordinated processes between angiogenesis and odontogenesis. The functional outcome has been described as “**vascularized dental pulp regeneration**” where appropriate. Furthermore, less precise expressions (e.g., “endothelial–odontogenic coupling”) have been replaced to ensure terminological consistency.

All corresponding changes have been implemented consistently throughout the manuscript and are highlighted in red.

**The Title (Line 2):**

“An Immunomodulatory Decellularized Pulp Matrix Hydrogel Promotes Vascularized Dental Pulp Regeneration Through Angiogenic-Odontogenic Coupling”

**The Abstract (Line 25, Line 31, Page1；Line 38, Page2):**

“…promote vascularized dental pulp regeneration.”

“…angiogenic–odontogenic paracrine interactions…”

“Taken together, our findings demonstrate that pDPM integrates immunomodulatory and angiogenic–odontogenic to support vascularized dental pulp regeneration, highlighting its potential as a next-generation biomaterial for clinical VPT.”

**The Keywords (Line 43, Page2):**

“Decellularized extracellular matrix, Angiogenic-odontogenic coupling, Macrophage polarization, Vascularized dental pulp regeneration, Pulp-capping materials, Dental pulp tissue engineering

**The Introduction (Line 120-124, Page6):**

“**Fig 1.** Schematic illustration of this study. pDPM hydrogel applied to exposed dental pulp promotes vascularized dental pulp regeneration. pDPM hydrogel derived from decellularized dental pulp regulates macrophage polarization via PI3K/AKT signaling, establishing a pro-regenerative immune microenvironment that enhances angiogenic–odontogenic coupling and promotes vascularized dental pulp regeneration.

**In Results (Section 3.2, Line 437, Page 22; Section 3.5, Line 595, Page 31; Section 3.7, Line 657-658, Page 35):**

“…supporting a pro-regenerative macrophage phenotype potentially conducive to vascularized dental pulp regeneration.

“…favorable for vascularized dental pulp regeneration.”

“…demonstrate that pDPM hydrogel promotes vascularized dental pulp regeneration in vivo by enhancing endothelial reconstruction and facilitating macrophage polarization toward a pro-regenerative M2 phenotype.”

**In Discussion (Line 769, Page 41):**

“… achieve functional vascularized dental pulp regeneration….”

**In Conclusion (Line 775, Page 41; Line 778-780, Page 41-42):**

“…. that orchestrates vascularized dental pulp regeneration.”

“…. which in turn enhanced angiogenic–odontogenic paracrine interactions. As a direct pulp capping material, pDPM-gel significantly enhanced vascularized dental pulp formation.”

*Comment 2*: In section 2.3, odontogenic induction generally requires the use of an induction medium. It is not appropriate to consider pDPM alone as an odontogenic induction condition. It is recommended that the authors supplement this section with relevant experiments.

*Response 2*:  We sincerely thank the reviewer for this insightful comment. We agree that odontogenic induction typically requires a defined differentiation medium, and it is not appropriate to consider pDPM alone as an odontogenic induction condition. We apologize that this methodological detail was not clearly described in the original manuscript, which may have led to misunderstanding.

In the revised manuscript, we have clarified the experimental design and supplemented the missing information in Section 2.3. Specifically, DPSCs in the lower chamber, which were used to evaluate odontogenic differentiation, were cultured in odontoblastic differentiation medium. This revision ensures that odontogenic differentiation was conducted under appropriate induction conditions, while pDPM primarily functioned as a modulatory cue in the co-culture system. The manuscript has been updated accordingly to improve clarity and rigor. The revised text is as follows and highlighted in red.

**In Materials and Methods (Section 2.3, Line 200-204, Page 10):**

“DPSCs were seeded in the lower chamber and were maintained in odontoblastic differentiation medium (α-MEM, 10% FBS, 50 μg/mL ascorbic acid, 10 mM β-glycerophosphate, and 100 nM dexamethasone) for subsequent evaluation of odontogenic differentiation.”

*Comment 3*: In section 2.4, the experimental details regarding macrophage polarization are not provided. Please supplement the specific experimental procedures.

*Response 3*: We sincerely thank the reviewer for this valuable comment. We apologize for the lack of detail. The experimental procedures for macrophage polarization have now been clearly and comprehensively described in the revised Section 2.4, including PMA-induced differentiation, pDPM treatment conditions, and subsequent marker analysis. The revised text is as follows and highlighted in red.

**In Materials and Methods (Section 2.4, Line 15-221, Page 10-11):**

“THP-1 derived macrophages were generated as described in Section 2.3. Briefly, THP-1 monocytes were induced with PMA (10 ng/mL) for 24 h, followed by 24 h culture in PMA-free medium. The differentiated macrophages were subsequently exposed to pDPM for 24 h to evaluate its effects on macrophage polarization. Classical M1 and M2 polarization markers (CD86 and Arg-1/CD206) were used to characterize the polarization status.”

*Comment 4*: In section 2.5, the heading reads "Protein analysis of DPM and its regulatory mechanisms on macrophage polarization," yet the experimental procedure does not include any content related to macrophage polarization.

*Response 4*: We sincerely thank the reviewer for this important comment. We agree that the original version of Section 2.5 primarily described the proteomic profiling of DPM. It lacked explicit experimental details regarding its regulatory mechanisms on macrophage polarization. This led to an inconsistency between the section title and the described methods.

In the revised manuscript, we have supplemented Section 2.5 with detailed experimental procedures investigating the underlying mechanism of DPM-mediated macrophage polarization. Specifically, we included PI3K/AKT pathway analysis and functional inhibition experiments using the PI3K inhibitor LY294002. The activation of AKT signaling (p-AKT) was assessed by western blotting, and the effects of pathway inhibition on macrophage polarization were evaluated by qRT-PCR and western blotting of M1 (CD86) and M2 (Arg-1) markers. These additions ensure that the experimental procedures are now consistent with the section title and support the conclusion that pDPM promotes macrophage M2 polarization via PI3K/AKT signaling. The revised text is provided below (highlighted in red).

**In Materials and Methods (Section 2.5, Line 249-256, Page 12):**

“To investigate the role of PI3K/AKT signaling in pDPM-mediated macrophage polarization, THP-1 derived macrophages were treated with pDPM in the presence or absence of LY294002 (10 μM). PI3K/AKT pathway activation was evaluated by western blotting of p-AKT and AKT after 24 h treatment. The involvement of PI3K/AKT signaling in macrophage polarization was further assessed by qRT-PCR analysis of CD86 and Arg-1. Protein expression was further evaluated by western blotting, with DMSO serving as the vehicle control.”

*Comment 5*: In section 2.10, please provide the specific cell culture conditions

*Response 5*: We sincerely thank the reviewer for this valuable comment. We agree that the specific cell culture conditions for qRT-PCR analysis were not sufficiently described in the original manuscript. In the revised manuscript, we have clarified the culture conditions associated with different experimental endpoints in Section 2.10. Specifically, cells were cultured under the Transwell co-culture conditions as described in Section 2.3. For odontogenic differentiation assays, DPSCs were maintained in odontoblastic differentiation medium for 14 days prior to RNA extraction. For angiogenic assays, HUVECs were cultured in DMEM containing 10% fetal bovine serum, 100 U/mL penicillin, and 100 μg/mL streptomycin under Transwell co-culture conditions for 3 days before analysis. These revisions clearly define the culture conditions corresponding to each time point and ensure the reproducibility of the experiments. The revised text is as follows (highlighted in red).

**In Materials and Methods (Section 2.10, Line 305-309, Page 15):**

“Cells were cultured under the Transwell co-culture conditions as described in Section 2.3. DPSCs were cultured in odontoblastic differentiation medium under Transwell co-culture conditions for 14 days prior to RNA extraction. For angiogenic assays, HUVECs were cultured under Transwell co-culture conditions for 3 days before analysis.”

*Comment 6*: In lines 351–353, please provide the reference for the statement: "Collagen I hydrogel, a widely used natural extracellular matrix - based biomaterial, was employed as a reference control for comparison with pDPM hydrogel.

*Response 6*: We sincerely thank the reviewer for this helpful comment. We agree that appropriate references should be provided to support the statement that collagen I hydrogel is a widely used natural extracellular matrix-based biomaterial. In the revised manuscript, we have added relevant high-impact references to substantiate this statement. The revised sentence now reads as follows (highlighted in red):

**In Materials and Methods (Section 2.14 Line 336-338, Page 16):**

“Collagen I hydrogel, a widely used natural extracellular matrix-based biomaterial, was employed as a reference control for comparison with pDPM hydrogel[24, 25].

24.	Hu N, Jiang R, Deng Y, Li W, Jiang W, Xu N, Wang J, Wen J, Gu S. Periapical lesion-derived decellularized extracellular matrix as a potential solution for regenerative endodontics. *Regen Biomater* 2024;11:rbae050.

25.	Zhu X, Yang Y, Mao S, Liu Q, Li Y, Yang Y, Gao M, Bao J, Li W, Li Y. Lung dECM matrikine-based hydrogel reverses bleomycin-induced pulmonary fibrosis by suppressing M2 macrophage polarization. *Biofabrication* 2024;17.”

*Comment 7*: Please provide an explanation for the use of chloral hydrate in rat anesthesia, clarify whether this was a writing error, and if chloral hydrate was indeed used, please provide the corresponding ethics approval document.

*Response 7*: We sincerely thank the reviewer for this important comment. We apologize for the confusion caused by the description of anesthesia in the original manuscript. The mention of chloral hydrate was a writing error. In the actual animal experiments, rats were anesthetized using Zoletil 50 (tiletamine–zolazepam), which is commonly used for laboratory animal anesthesia due to its safety and reliability. We have corrected this information in the revised manuscript. The animal experiments were conducted in accordance with institutional guidelines and were approved by the Animal Ethics Committee of Sun Yat-sen University, with the approval number updated as SYSU-IACUC-2026-001539. The relevant description has been revised accordingly in the manuscript (highlighted in red).

**In Materials and Methods (Section 2.14 Line 338-339 Page 16):**

“Following anesthesia with Zoletil 50, ...”

**In Materials and Methods (Line 127-129, Page 6):**

“All procedures involving animals were reviewed and authorized by the Animal Care and Use Committee of Sun Yat-sen University (Approval No. SYSU-IACUC-2026-001539).”

*Comment 8*: The gelation time of pDPM is 30 minutes. Please specify the timing for applying the glass ionomer seal and discuss in the Discussion section whether this hydrogel can achieve a reliable coronal seal in combination with the glass ionomer.

*Response 8*: We sincerely appreciate this important comment. We agree that the gelation behavior of pDPM is highly relevant to its practical intraoperative handling and subsequent coronal sealing.

To further clarify the handling properties of pDPM hydrogel, we have supplemented rheological analysis (time sweep) in the revised manuscript. The results show that upon rapid temperature increase from 4°C to 37°C, the storage modulus (G’) of pDPM hydrogels increased markedly, and reached a plateau within approximately 350 seconds (∼5 minutes), indicating the establishment of a stable gel network. Based on this result, in the animal procedure, the hydrogel was allowed to undergo initial gelation for approximately 5 minutes prior to the placement of glass ionomer cement (GIC), which is sufficient to ensure material retention at the exposure site. Although full gelation required additional time, the material exhibited sufficient viscosity and early-stage structural integrity after placement to remain within the confined pulp space prior to complete gelation. Importantly, pDPM is intended to function as an intracavitary regenerative matrix beneath the coronal restoration rather than as the primary sealing material. Therefore, the definitive coronal seal and mechanical protection was provided by the overlying GIC, while pDPM served as a bioactive scaffold within the pulp chamber. This combination strategy is consistent with current clinical practice in vital pulp therapy.

These details have now been clarified in the Methods, Results and Discussion sections (highlighted in red).

**In Materials and Methods (Section 2.2 Line 171-178, Page 8-9):**

“Rheological properties of pDPM-gel were characterized with a rheometer (TA Instruments, USA). Frequency sweep tests were performed at 37 °C under 1% strain. Temperature and time sweep analyses were performed to assess the sol–gel transition and gelation kinetics, with temperature was increased from 4 to 40 °C at 3 °C/min and time sweep conducted at 37 °C under 1% strain and 1 Hz. Viscosity measurements were carried out over at shear rate ranging from 0.1–500 s⁻¹ to assess shear-thinning behavior.”

**In Results (Section 3.1, Line 371-407, Page 18-20):**

“To further evaluate the physicochemical properties of pDPM hydrogel, rheological analyses were performed. Temperature sweep analysis demonstrated that pDPM hydrogel exhibited thermoresponsive gelation behavior, with the storage modulus (G’) increasing markedly as the temperature rose from 4 °C to 37 °C and surpassing the loss modulus (G’’), indicating a transition from a sol to a gel state (Fig 2O). Time sweep analysis at 37 °C revealed rapid gelation kinetics, with G’ increasing sharply and reaching a plateau within approximately 300–400 s (∼5 min), suggesting the establishment of a stable viscoelastic network (Fig 2P). Viscosity measurements further showed that pDPM-gel exhibited typical shear-thinning property, supporting its injectability for minimally invasive delivery (Fig 2Q). In addition, frequency sweep analysis confirmed the viscoelastic stability of the hydrogel, with G’ consistently higher than G’’ across the tested frequency range, indicating a stable gel structure (Fig 2R).

**Fig 2.** … (O) Temperature sweep showing thermoresponsive gelation of pDPM hydrogel (G’: storage modulus; G’’: loss modulus). (P) Time sweep showing rapid gelation kinetics (G’ > G’’). (Q) Viscosity measurement showing shear-thinning behavior. (R) Frequency sweep showing stable viscoelastic properties (G’ > G’’).”

**In Discussion (Line 689-699, Page 37-38):**

“The pDPM hydrogel further recapitulated this structural within a three-dimensional matrix environment while exhibiting thermoresponsive gelation and shear-thinning behavior, enabling injectability and stable retention within the pulp cavity. In the in vivo procedure, the hydrogel was allowed to undergo initial gelation for approximately 5 min before placement of the overlying glass ionomer, which was sufficient to support material retention at the exposure site. Rather than serving as a load bearing or sealing material, the hydrogel functions as a viscoelastic intracavitary matrix that adapts closely to cavity walls and supports the sealing performance of the overlying restoration. These properties suggest that pDPM is structurally compatible with current clinical protocols in VPT while providing additional biological functionality [37, 38].”

*Comment 9*: The specific gelation process of pDPM is critical for in vivo application. In Figure 2, please provide an illustration of the gelation process of pDPM

*Response 9*: Thank you for this valuable suggestion. We agree that visualization of the gelation process is important for understanding the in vivo applicability of pDPM hydrogel. In response, we have added a schematic illustration in the revised Figure 2 to depict the sol–gel transition process of pDPM.

Briefly, pDPM remains in a fluid state at low temperature (4 °C), allowing for facile injection into the pulp cavity. Upon exposure to physiological temperature (37 °C), the hydrogel undergoes a sol to gel transition and forms a stable three-dimensional network. This schematic representation has been incorporated into Figure 2, and the corresponding description has been added to the figure legend and Results section to improve clarity. The revised text is as follows and highlighted in red.

**In Results (Section 3.1, Line 367-370, Page 17-18; Line 400-401, Page 20):**

“In addition, a schematic illustration was provided to depict the sol to gel transition process of pDPM, showing its injectable fluid state at low temperature and gel formation at physiological temperature (Fig 2N).”

**Fig 2. (N)** Schematic illustration of the sol to gel transition process of pDPM under physiological conditions.

*Comment 10*: The language of the manuscript requires further polishing. Noun usage should remain consistent; for example, "DMP-1" and "DMP1" appear inconsistently throughout the text and should be unified.

*Response 10*: Thank you for this helpful comment. We have carefully revised the manuscript to ensure consistency in terminology throughout the text. Specifically, the nomenclature for dentin matrix protein 1 has been unified as “DMP1” across the manuscript. In addition, we have systematically standardized the naming of all genes, proteins, and biomaterials (e.g., DMP1, Arg-1, and pDPM-gel) to ensure consistency. The manuscript has also been thoroughly edited for language, grammar, and readability to improve overall clarity. All corresponding changes have been implemented throughout the manuscript and are highlighted in red. Representative revisions are provided below:

**In Materials and Methods (Section 2.10, Line 303, Page 14; Section 2.11, Line 312, Page 15):**

“Gene expression levels associated with odontoblastic differentiation (*DMP1, DSPP, ALP,* and *RUNX2*) at 14 days …”

“Protein expression levels of odontogenic markers (DMP1, DSPP, ALP...”

**In Materials and Methods (Section 2.1, Line 132, 146, Page 7)**

“*2.1 Fabrication of DPM and pDPM hydrogel (pDPM-gel)*

…To prepare pDPM-gel,…”

**In Results (Section 3.1, Line 364, Page 17):**

“Moreover, the pDPM hydrogel (pDPM-gel) exhibited a nanofibrous architecture with fiber morphology and diameter distribution like those of Col I-gel (Fig 2K, L).”

*Comment 11*: In Figure 7c, a clear dental pulp tissue structure is not observed in the experimental group. Please supplement this content.

*Response 11*: Thank you for this important comment. We agree that the dental pulp tissue structure was not clearly visible in the original Figure 7c. Upon careful re-examination, we found that this was due to partial tissue detachment during the sectioning and staining process, which affected the preservation of pulp tissue in that specific section. To address this issue, we have replaced the image of the Col-gel and pDPM-gel group in Figure 7c with a representative section in which the pulp tissue is well preserved and clearly visible. The revised image better reflects the histological features of the regenerated tissue. This update does not alter the overall findings or conclusions of the study. The corresponding figure and description have been updated in the revised manuscript (highlighted in red).

**In Results (Section 3.6, Line 632, Page 34):**

*Comment 12*: Please explain the reason for the appearance of SHEDs cells in the supplementary files.

*Response 12*: Thank you for this careful observation. We apologize for the confusion caused by the appearance of “SHEDs” in the supplementary files. Upon verification, this was an inadvertent labeling error. The cells used in this study were dental pulp stem cells (DPSCs), rather than stem cells from human exfoliated deciduous teeth (SHEDs). We have corrected all instances of “SHEDs” to “DPSCs” in the supplementary materials and have carefully reviewed the entire manuscript to ensure consistency in cell nomenclature throughout. The source and characteristics of DPSCs used in this study remain unchanged and are accurately described in the Methods section. This correction does not affect the experimental results or the conclusions of the study. The revised text is as follows and highlighted in red.

**In Supplementary Materials**

“**Figure S1.** Characterization and proliferation of DPSCs. 3 days of primary DPSCs (A) and DPSCs at P3 (B).”

*Comment 13*: In the context of pulpitis, inflammation control is a prerequisite for achieving dental pulp tissue repair. Please supplement experiments assessing the expression of inflammatory cytokines.

*Response 13*: Thank you for this valuable comment. We agree that inflammation control is a prerequisite for successful dental pulp repair. In response to this comment, we have supplemented additional experiments to evaluate inflammatory responses under simulated inflammatory conditions. Specifically, an LPS-induced inflammatory macrophage model was established, and the expression of inflammatory markers were evaluated. As shown in the revised manuscript, pDPM treatment significantly reduced pro-inflammatory marker expression (iNOS) while increasing anti-inflammatory markers (CD206) and cytokine expression (IL-10) compared with the LPS group. These findings provide direct evidence that pDPM modulates inflammatory responses and promotes a pro-regenerative immune microenvironment under inflammatory conditions. The corresponding data have been added to the revised manuscript (highlighted in red).

**In Materials and Methods (Section 2.4, Line 210, Page 10; Line229-235, Page 11)**

*“2.4 Macrophage polarization, inflammatory modulation, and angiogenic–odontogenic factor analysis*

“For inflammatory modulation analysis, THP-1–derived macrophages were first stimulated with LPS (100 ng/mL) for 12 h, followed by pDPM treatment for 24 h. Immunofluorescence staining was used to assess iNOS and CD206 using primary antibodies diluted at 1:200 (Affinity, China). qRT-PCR was conducted to determine the expression level of *iNOS* and *IL-10*. Protein levels of iNOS and IL-10 were assessed by western blotting using primary antibodies diluted at 1:1000 (Affinity, China).”

**In Results (Section 3.2, Line 424-432, Page 21; Line 438, Page 22; Line448-453, Page 23)**

“To further evaluate whether pDPM retains its immunomodulatory activity under inflammatory microenvironment, an LPS-stimulated macrophage model was generated. Immunofluorescence staining demonstrated that pDPM markedly reduced iNOS expression while increasing CD206 expression compared with the LPS group (Fig. 3F, H). qRT-PCR analysis showed decreased iNOS levels, together with increased IL-10 expression following pDPM treatment (Fig. 3G). Western blot analysis further confirmed reduced iNOS protein expression and elevated IL-10 levels in the pDPM-treated group (Fig. 3I).

Fig3. …(F) Representative immunofluorescence staining of CD206 (green) and iNOS (red) expression in Control, LPS, and LPS + pDPM groups. (G) qRT-PCR evaluate of *iNOS* and *IL-10* mRNA expression normalized to GAPDH. (H) Semi-quantitative analysis of iNOS and CD206 fluorescence intensity. (I) Western blotting and densitometric analysis of iNOS and IL-10.”

**In Supplementary Materials (Table S1)**

| Target gene | Primer sequences |
| --- | --- |
| *iNOS* | Forward: GAGCCAGGCCACCTCTATGT |
|  | Reverse: GTCCTCGACCTGCTCCTCAT |
| *IL-10* | Forward: GACTTTAAGGGTTACCTGGGTTG |
|  | Reverse: TCACATGCGCCTTGATGTCTG |

**Response to Reviewer #2**

*Comment：*This study focuses on a porcine-derived decellularized pulp matrix hydrogel (pDPM-gel), aiming to explore its potential in promoting vascularized dentin regeneration in pulpotomy procedures. While the paper conceptually proposes an innovative "immunomodulation-angiogenesis-dentinogenesis" triad mechanism, it exhibits notable deficiencies in the characterization of physicochemical properties, functional validation of vasculature, and the rigor of clinical translation. Without a major revision, this paper should not be under consideration for publication at this stage.

*Response*: Thank you for your precious time to review our manuscript. We highly appreciate your valuable comments regarding the physicochemical characterization of the hydrogel, the evaluation of its immunomodulatory function under inflammatory conditions, and the considerations on translational relevance. We acknowledge that these issues are critical for evaluating the functionality and clinical potential of pDPM-gel. In response, we have carefully revised the manuscript and, where possible, supplemented additional experimental data and analyses to address these concerns. Specifically, we have strengthened the physicochemical characterization, further evaluated the immunomodulatory performance under inflammatory conditions, and clarified the limitations and translational implications of our study. We believe that these revisions have substantially improved the scientific rigor and completeness of the work. We hope that the revised manuscript will better meet the standards for publication.

Detailed point-by-point responses to each of your comments are provided below.

*Comment 1*: The manuscript only shows the microstructure under SEM (Figure 2K, L), but lacks the most critical rheological properties data for a hydrogel. For an injectable hydrogel, data on Storage Modulus (G'), Loss Modulus (G''), gelation time, temperature/pH sensitivity, and mechanical strength are mandatory. Without this data, it is impossible to judge whether the material can maintain structural stability under the humid environment of the oral cavity and masticatory pressure, failing to support the clinical claim of "minimally invasive intervention.

*Response 1*: We sincerely appreciate this valuable comment. We fully agree that comprehensive rheological characterization is critical for evaluating the clinical applicability and structural reliability of injectable hydrogels, particularly in the oral environment. To address this concern, we have supplemented systematic rheological analyses of pDPM-gel at the different concentration in the revised manuscript, including **storage modulus (G′) and loss modulus (G″), gelation kinetics (time sweep), temperature-dependent sol–gel transition (temperature sweep), frequency sweep, and shear viscosity measurements**. The results demonstrated that pDPM-gel exhibited favorable thermoresponsive gelation behavior, rapid gelation kinetics, and shear-thinning properties suitable for injectable delivery. In addition, the storage modulus (G') exceeded the loss modulus (G'') after gelation, indicating the formation of a stable viscoelastic network.

We thank the reviewer for highlighting the importance of pH sensitivity under inflammatory conditions. In the present study, our rheological evaluation focused on the thermoresponsive gelation behavior that is most directly related to injectable handling and in situ gel formation. We agree that pH-dependent properties may also be relevant to the biological microenvironment and will be investigated separately in future studies.

Regarding mechanical strength, we agree that structural stability is important for maintaining space and supporting tissue regeneration. In the context of vital pulp therapy, pDPM-gel is applied within the confined pulp chamber and protected by a coronal restorative material (e.g., glass ionomer cement). Therefore, its primary mechanical requirements relate to maintaining structural integrity and resisting deformation in a hydrated and confined environment, which is partially supported by its stable viscoelastic behavior and rapid gelation in rheological analyses, rather than directly bearing occlusal forces. Direct mechanical testing, such as compressive modulus measurement, was not included in the present study; however, we agree that this is an important aspect and will address it in future work.

Taken together, these rheological properties—particularly injectability, rapid in situ gelation, and viscoelastic stability—support the feasibility of minimally invasive delivery through syringe-based application. The corresponding results have been added to the revised manuscript and highlighted in red.

**In Materials and Methods (Section 2.2, Line 171-178, Page 8-9):**

“Rheological properties of pDPM-gel were characterized with a rheometer (TA Instruments, USA). Frequency sweep tests were performed at 37 °C under 1% strain. Temperature and time sweep analyses were performed to assess the sol–gel transition and gelation kinetics, with temperature was increased from 4 to 40 °C at 3 °C/min and time sweep conducted at 37 °C under 1% strain and 1 Hz. Viscosity measurements were carried out over at shear rate ranging from 0.1–500 s⁻¹ to assess shear-thinning behavior.”

**In Results (Section 3.1, Line 371-384, Page 18; Line 385, Page 19; Line 401-406, Page 20):**

“To further evaluate the physicochemical properties of pDPM hydrogel, rheological analyses were performed. Temperature sweep analysis demonstrated that pDPM hydrogel exhibited thermoresponsive gelation behavior, with the storage modulus (G’) increasing markedly as the temperature rose from 4 °C to 37 °C and surpassing the loss modulus (G’’), indicating a transition from a sol to a gel state (Fig 2O). Time sweep analysis at 37 °C revealed rapid gelation kinetics, with G’ increasing sharply and reaching a plateau within approximately 300–400 s (∼5 min), suggesting the establishment of a stable viscoelastic network (Fig 2P). Viscosity measurements further showed that pDPM-gel exhibited typical shear-thinning property, supporting its injectability for minimally invasive delivery (Fig 2Q). In addition, frequency sweep analysis confirmed the viscoelastic stability of the hydrogel, with G’ consistently higher than G’’ across the tested frequency range, indicating a stable gel structure (Fig 2R)

**Fig 2.** …(O) Temperature sweep showing thermoresponsive gelation of pDPM hydrogel (G’: storage modulus; G’’: loss modulus). (P) Time sweep showing rapid gelation kinetics (G’ > G’’). (Q) Viscosity measurement showing shear-thinning behavior. (R) Frequency sweep showing stable viscoelastic properties (G’ > G’’).”

**In Discussion (Line 768-699, Page 37-38):**

“The pDPM hydrogel further recapitulated this structural within a three-dimensional matrix environment while exhibiting thermoresponsive gelation and shear-thinning behavior, enabling injectability and stable retention within the pulp cavity. In the in vivo procedure, the hydrogel was allowed to undergo initial gelation for approximately 5 min before placement of the overlying glass ionomer, which was sufficient to support material retention at the exposure site. Rather than serving as a load bearing or sealing material, the hydrogel functions as a viscoelastic intracavitary matrix that adapts closely to cavity walls and supports the sealing performance of the overlying restoration. These properties suggest that pDPM is structurally compatible with current clinical protocols in VPT while providing additional biological functionality [37, 38].”

*Comment 2*: Pulp repair occurs in a complex inflammatory microenvironment. Macrophages induced solely by PMA cannot simulate the cellular response under pulpitis conditions. There is a lack of experiments verifying the immunomodulatory capacity of pDPM under inflammatory conditions (e.g., LPS stimulation).

*Response 2*: We sincerely appreciate this insightful comment. We fully agree that pulp repair occurs within a complex inflammatory microenvironment, and PMA-induced macrophages alone cannot fully recapitulate the pathological conditions of pulpitis. To address this important concern, we performed additional experiments using an inflammatory macrophage model established by LPS stimulation following PMA differentiation of THP-1 cells. This model better mimics the inflammatory microenvironment associated with pulpitis.

Immunofluorescence staining, Western blotting, and qPCR analyses were conducted to evaluate the immunomodulatory effects of pDPM under inflammatory conditions. The results showed that pDPM modulated inflammation, with reduced M1 marker (iNOS) expression and increased M2 markers (CD206 and IL-10) expression. These findings indicate that pDPM retains its immunomodulatory effects under inflammatory conditions, suggesting its potential application for pulp repair under pathological conditions such as pulpitis. The corresponding methods and results have been added to the revised manuscript (highlighted in red).

**In Materials and Methods (Section 2.4, Line 210, Page 10; Line 229-235, Page 11)**

*“2.4 Macrophage polarization, inflammatory modulation, and angiogenic–odontogenic factor analysis*

For inflammatory modulation analysis, THP-1–derived macrophages were first stimulated with LPS (100 ng/mL) for 12 h, followed by pDPM treatment for 24 h. Immunofluorescence staining was used to assess iNOS and CD206 using primary antibodies diluted at 1:200 (Affinity, China). qRT-PCR was conducted to determine the expression level of *iNOS* and *IL-10*. Protein levels of iNOS and IL-10 were assessed by western blotting using primary antibodies diluted at 1:1000 (Affinity, China)..”

**In Results (Section 3.2, Line 424-432, Page 21; Line 438, Page 22; Line 449-452, Page 23)**

“To further evaluate whether pDPM retains its immunomodulatory activity under inflammatory microenvironment, an LPS-stimulated macrophage model was generated. Immunofluorescence staining demonstrated that pDPM markedly reduced iNOS expression while increasing CD206 expression compared with the LPS group (Fig. 3F, H). qRT-PCR analysis showed decreased *iNOS* levels, together with increased *IL-10* expression following pDPM treatment (Fig. 3G). Western blot analysis further confirmed reduced iNOS protein expression and elevated IL-10 levels in the pDPM-treated group (Fig. 3I)

Fig3. …(F) Representative immunofluorescence staining of CD206 (green) and iNOS (red) expression in Control, LPS, and LPS + pDPM groups. (G) qRT-PCR evaluate of *iNOS* and *IL-10* mRNA expression normalized to GAPDH. (H) Semi-quantitative analysis of iNOS and CD206 fluorescence intensity. (I) Western blotting and densitometric analysis of iNOS and IL-10.”

**In Supplementary Materials (Table S1)**

| Target gene | Primer sequences |
| --- | --- |
| *iNOS* | Forward: GAGCCAGGCCACCTCTATGT |
|  | Reverse: GTCCTCGACCTGCTCCTCAT |
| *IL-10* | Forward: GACTTTAAGGGTTACCTGGGTTG |
|  | Reverse: TCACATGCGCCTTGATGTCTG |

*Comment 3*: Although LC-MS/MS analysis was performed (Figure 4), it only maintained GO and KEGG enrichment analysis. There is a lack of absolute quantification of key growth factors (such as VEGF, FGF-2, BMPs). Without knowing the specific concentration of these key factors, it is impossible to determine whether the biochemical factors inherent in the material are at work, or if it is merely the physical scaffold inducing cell behavior.

*Response 3*: We sincerely appreciate this insightful comment. We agree that quantitative characterization of retained bioactive factors would provide additional mechanistic information regarding the regenerative activity of pDPM. In the present study, our LC-MS/MS analysis was primarily used to characterize the global protein composition of pDPM and identify enriched biological pathways. We acknowledge that this approach does not provide absolute quantification of specific growth factors such as VEGF, FGF-2, and BMPs. Furthermore, we agree that without quantitative data, it remains difficult to fully distinguish the relative contributions of biochemical cues versus structural scaffold effects.

Nevertheless, the biological activity of pDPM was supported by functional evidence, including its ability to modulate macrophage polarization and enhance angiogenic and odontogenic responses in both in vitro and in vivo models. These findings suggest that pDPM retains bioactive properties relevant to tissue regeneration. As decellularized extracellular matrix materials exert bioactivity through multiple coordinated components, including matrix-bound proteins, cryptic peptides, glycosaminoglycans, and structural ligands, their regenerative effects are not solely dependent on the concentration of a few soluble growth factors.

We agree that targeted quantification of specific factors would be valuable and plan to pursue this in future studies using ELISA or multiplex assays. The corresponding discussion has been added to the revised manuscript (highlighted in red).

**In Discussion (Line 706-711, Page 38):**

“Although targeted quantification of individual growth factors was not performed, the regenerative activity of pDPM may be mediated by the combined effects of retained matrix-associated proteins, glycosaminoglycans, structural ligands, and tissue-specific microenvironmental cues rather than isolated soluble factors alone [43, 44].”

*Comment 4*: Although rats have a less severe immune rejection to porcine materials than humans, the manuscript does not detail the detection of host systemic immune reaction indicators. Moreover, using human cells (THP-1, HUVECs, DPSCs) with porcine materials in vitro presents issues of species-specific factor mismatch (Cross-reactivity), which may overestimate or underestimate the actual bioactivity of the material.

*Response 4*: Thank you for this important and insightful comment. We fully agree that both host immune response and potential cross-species effects are critical considerations for evaluating the translational relevance of porcine-derived biomaterials. Regarding the in vivo immune response, we have supplemented additional data to address this concern. Histological analysis of major organs (heart, liver, spleen, lung, and kidney) has been performed and is now included in the Supplementary Information. **Following subcutaneous injection of pDPM hydrogel in mice**, no obvious histopathological abnormalities or tissue damage were observed, suggesting preliminary in vivo biosafety of the material.

Regarding the potential issue of cross-species interactions in vitro, we acknowledge that the use of human-derived cells (THP-1, HUVECs, and DPSCs) in combination with porcine-derived matrix may introduce variability. However, a key advantage of decellularized extracellular matrix materials is that cellular and immunogenic components are largely removed, while structurally and functionally conserved ECM proteins and bioactive molecules are retained. These components, including collagen, laminin, and many growth factors, are relatively conserved across species and have been widely reported to exhibit cross-species bioactivity [1, 2]. Therefore, although species-specific differences cannot be completely excluded, the use of human cells remains a commonly accepted approach to evaluate the biological functions and mechanisms of biomaterials in vitro [3,4]. Importantly, our in vivo results in a rat model further support the biocompatibility and regenerative potential of pDPM in a living system. We acknowledge this limitation and will consider employing species-matched models or more clinically relevant systems in future studies to further validate the translational applicability of this material. The corresponding data have been added to the revised manuscript (highlighted in red).

**In Materials and Methods (Line 127-131, Page 6)**

“All procedures involving animals were reviewed and authorized by the Animal Care and Use Committee of Sun Yat-sen University (Approval No. SYSU-IACUC-2026-001539 and SYSU-IACUC-2026-001920). All in vivo studies were performed in compliance with the National Institutes of Health (NIH) Guidel for the Care and Use of Laboratory Animals”

**In Materials and Methods (Section 2.13, Line 324-328, Page 15-16)**

*2.13 In vivo biosafety evaluation by subcutaneous injection in mice*

“To evaluate the in vivo biosafety of pDPM hydrogel, healthy male mice (6 to 8 weeks old) were used. The pDPM hydrogel precursor solution was administered subcutaneously to the mice. At 7 days after injection, major organs were harvested for histological evaluation.”

**In Results (Section 3.7, Line 660-663, Page 36) and In Supplementary Materials (Fig S2):**

“In addition, no evident pathological abnormalities were observed in major organs at 7 days after subcutaneous injection of pDPM-gel in mice, indicating no obvious histopathological abnormalities in major organs at the examined time point (Fig. S2).

**Fig S2.** Histological evaluation of major organs following subcutaneous injection of pDPM hydrogel in mice.”

1.	Liu J, Song Q, Yin W, Li C, An N, Le Y, Wang Q, Feng Y, Hu Y, Wang Y. Bioactive scaffolds for tissue engineering: a review of decellularized extracellular matrix applications and innovations. In. *Exploration*: Wiley Online Library; 2025, 20230078.

2.	Kasravi M, Ahmadi A, Babajani A, Mazloomnejad R, Hatamnejad MR, Shariatzadeh S, Bahrami S, Niknejad H. Immunogenicity of decellularized extracellular matrix scaffolds: a bottleneck in tissue engineering and regenerative medicine. *Biomaterials research* 2023;27:10.

3.	Kim YJ, Hong S, Lee JH, Lee J, Kang JJ, Bae J, Kim J, Kim DH, Yoo J, Cha MS, Kim SH, Jung Y. Amine-Functionalized Chitosan/dECM Composite Promotes Cutaneous Regeneration and Hair Follicle Activation by Regulating Oxidative Stress and Inflammation. *Adv Healthc Mater* 2026;15:e04383.

4.	Peng P, Fan S, Liu P, Huang X, Xuan Z, Liu A, Wu M, Sui B, Ye B, Zhang S, Guo H. Porcine decellularized splenic matrix hydrogel-based vesicle sustained-release System: A dual-regulatory biomaterial for accelerated wound healing via angiogenesis and immune remodeling. *Mater Today Bio* 2025;35:102601.

Thank you again for your time and consideration. We sincerely appreciate the constructive comments and suggestions, which have helped us improve the quality and clarity of our manuscript. We hope that the revised version is now suitable for publication in ***Regenerative Biomaterials***. Please do not hesitate to contact us if any further revisions are required.
